# Non-Surgical Interventions for Adolescents with Idiopathic Scoliosis: An Overview of Systematic Reviews

**DOI:** 10.1371/journal.pone.0110254

**Published:** 2014-10-29

**Authors:** Maciej Płaszewski, Josette Bettany-Saltikov

**Affiliations:** 1 Faculty of Physical Education and Sport in Biała Podlaska, University School of Physical Education, Warsaw, ul. Akademicka 2, Biała Podlaska, Poland; 2 University of Teesside, School of Health and Social Care, Middlesbrough, United Kingdom; Griffith University, Australia

## Abstract

**Background:**

Non-surgical interventions for adolescents with idiopathic scoliosis remain highly controversial. Despite the publication of numerous reviews no explicit methodological evaluation of papers labeled as, or having a layout of, a systematic review, addressing this subject matter, is available.

**Objectives:**

Analysis and comparison of the content, methodology, and evidence-base from systematic reviews regarding non-surgical interventions for adolescents with idiopathic scoliosis.

**Design:**

Systematic overview of systematic reviews.

**Methods:**

Articles meeting the minimal criteria for a systematic review, regarding any non-surgical intervention for adolescent idiopathic scoliosis, with any outcomes measured, were included. Multiple general and systematic review specific databases, guideline registries, reference lists and websites of institutions were searched. The AMSTAR tool was used to critically appraise the methodology, and the Oxford Centre for Evidence Based Medicine and the Joanna Briggs Institute’s hierarchies were applied to analyze the levels of evidence from included reviews.

**Results:**

From 469 citations, twenty one papers were included for analysis. Five reviews assessed the effectiveness of scoliosis-specific exercise treatments, four assessed manual therapies, five evaluated bracing, four assessed different combinations of interventions, and one evaluated usual physical activity. Two reviews addressed the adverse effects of bracing. Two papers were high quality Cochrane reviews, Three were of moderate, and the remaining sixteen were of low or very low methodological quality. The level of evidence of these reviews ranged from 1 or 1+ to 4, and in some reviews, due to their low methodological quality and/or poor reporting, this could not be established.

**Conclusions:**

Higher quality reviews indicate that generally there is insufficient evidence to make a judgment on whether non-surgical interventions in adolescent idiopathic scoliosis are effective. Papers labeled as systematic reviews need to be considered in terms of their methodological rigor; otherwise they may be mistakenly regarded as high quality sources of evidence.

**Protocol registry number:**

CRD42013003538, PROSPERO

## Introduction

Non-surgical interventions for the treatment of adolescents with idiopathic scoliosis in current practice today typically constitute a variety of physical modalities; these include braces, scoliosis-specific exercises as well as diverse physical therapy modalities such as manual therapy and electrical stimulation [1]–[5]. Other forms of non-surgical therapies reported in the literature include podiatric treatments such as heel lifts as well as different types of osteopathic and chiropractic interventions. Additionally, complementary and alternative interventions have also been reported [6]–[8]. Non-surgical interventions for adolescents with idiopathic scoliosis as a whole remains a contentious issue, with conflicting recommendations put forward from clinical research studies and as well as experts in the field. Interestingly authors have reported both very negative as well as very positive statements (Table 1).

**Table 1:** Opinions regarding non-surgical interventions for adolescents with idiopathic scoliosis.

**negative comments:**
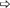 “time and common sense prevent me from discussing any other treatment modality than bracing”[9]
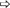 “treatment options for patients with scoliosis range from the unproven or harmful to the beneficial” [10]
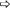 “physical therapy, chiropractic care, biofeedback and electric stimulation have not been shown to alter the natural history of scoliosis” [11]
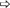 “patients should be aware of the absence of evidence for these [physiotherapy] treatments” [12]
**positive statements:**
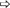 “bracing and spinal surgery have been proven to alter the natural history of curve progression” [13]
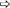 “exercise-based therapies, alone or in combination with orthopaedic approaches, are a logical approach to improve and maintain flexibility and function in patients at risk for pain, pulmonary dysfunction, and progression” [14]
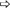 “the triad of out-patient physiotherapy, intensive in-patient rehabilitation and bracing has proven effective in conservative scoliosis treatment in central Europe” [15]

The statements above reflect the clinical equipoise currently represented by surgeons, physicians, physical therapists and other health care professionals to the non-surgical treatment approaches of AIS, especially regarding scoliosis-specific exercise treatments (SSEs). These interventions, defined as “curve-specific movements performed with the therapeutic aim of reducing the deformity” [16], consist of individually adapted exercises that are taught to patients in a centre that is totally dedicated to scoliosis treatment. The patients learn an exercise protocol that is personalized according to medical and physiotherapeutic evaluations. SSEs have traditionally been used in continental Europe by different specialized scoliosis centers or “schools” [1], either as a sole treatment or supplementing orthotic brace treatment [17], [18]. Further as stated above, other types of non-surgical interventions reported in the literature include manual therapies [6], [7] different types of chiropractic and osteopathic interventions as well as numerous unorthodox complementary and alternative forms of treatments [8] have been applied to different patient groups in different contexts.

Physiotherapy interventions are typically not regarded as effective in Anglo-Saxon countries [1], [2], despite the fact that the evidence-base for the inefficacy of exercise treatments seems questionable [2]. Bracing meanwhile has been recommended as the standard treatment [1], [3], [19]–[21], despite a weak evidence-base being reported [4], [22] prior to the latest and very recent publication from a multicenter controlled trial [23]. The general recommendations on the non-surgical management of AIS [5], [10], [21] put forwards by the different scoliosis societies [3], [24], tend to contain conflicting information and generally do not distinguish between different approaches and types of braces, as well as between the use of rigid and soft braces [4], [25], [26]. The physiotherapists’ role is typically seen by surgeons and physicians as complementary to the multidisciplinary team that cares for braced patients [27]. Nonetheless, the interest in scoliosis-specific exercise interventions has in recent years become more widespread, with the availability of thematic issues within healthcare journals relevant to spinal conditions [28], [29], courses on the PT management of scoliosis becoming increasingly available as well as high profile RCTs currently being funded and conducted in the United Kingdom [30], Canada [31], and Sweden [32].

### Why is this overview of systematic reviews needed?

In view of the existing prejudices and considerable variations in recommendations [3], [24] and opinions, both between and within different professional groups, especially with regards to the effectiveness of bracing, as opposed to the merits of SSE and other non-surgical forms of interventions, systematic reviews remain important sources of evidence for all engaged in AIS therapy.

In recent years two Cochrane reviews [16], [33] several other systematic reviews (SRs) (PEDro database indexed 17 SRs in April 2014) as well as papers labeled as “evidence-based” have been published (Tables S1 and S2). These have included the measurement of numerous outcome measures as well as different inclusion criteria and study designs, with each review reaching different conclusions. The effectiveness of non-surgical interventions for the treatment of adolescents with idiopathic scoliosis remains highly controversial with the evidence-base for informing service users, practitioners and stakeholders confusing and unclear.

Within the existing literature (with the exception of a few structured abstracts provided by the DARE database) the authors were unable to find any high quality methodological evaluations of published SRs. The latter were either accepted at face value [29], [34], [35] criticized without further explicit analyses [36], [37] or the results were discussed only in terms of the research designs of included studies [16], [33].

Even on initial reading of the available SRs it appeared that large and significant differences with regards to the way they were conducted i.e. their methodological quality were present. It is important to consider that not ALL papers labeled as “systematic” or “evidence-based” actually DO meet the criteria for a systematic review. These inconsistencies strongly suggested that a comprehensive and systematically undertaken methodological analysis of currently published systematic reviews addressing the non-surgical management of AIS was urgently needed and warranted.

### Objectives

The primary objective of this study was to provide a comprehensive and systematic analysis of the scope, objectives, methodology and findings from published SRs regarding non-surgical interventions of AIS, through conducting an overview of systematic reviews.

The second objective was to establish, which papers currently labeled as “systematic reviews” or having the layout of a systematic review did NOT on further analysis meet the minimal criteria for a SR, and were in fact opinion based papers rather than well conducted secondary research studies.

Finally the third objective was to analyse and compare findings from different SRs addressing the same types of interventions, to enable judgments to be made regarding the evidence-base for their use within clinical practice.

## Materials and Methods

This paper reports on a section of an overview of systematic reviews evaluating the effectiveness of non-surgical management for adolescent idiopathic scoliosis, including screening and treatment methods, and is registered at PROSPERO, CRD York, CRD42013003538 (Protocol S1).

The PRISMA statement for undertaking and reporting systematic reviews [38], [39] was followed. Further the proposal for the applicability of the PRISMA statement items for overviews of systematic reviews was consulted and adhered to [40].

### Criteria for inclusion of systematic reviews

#### Study designs

Systematic reviews were considered eligible if they included primary papers of any types of experimental and observational study designs. These liberal criteria were introduced in order to allow the authors to evaluate all published SRs addressing the subject matter.

Papers were reported as systematically developed reviews if they reported on methods to search, identify and select papers, and critically appraised relevant evidence [41]. If found, these minimal criteria were also applied to reviews of evidence, prepared for or reported in, systematically developed clinical practice guidelines and recommendations, on the condition that they were reported in full. **Exclusion criteria** were; reports from any types of primary studies, expert opinions, narrative reviews and other types of non-systematic reviews (e.g. critical reviews), letters to the editor and editorials. Systematic overviews of reviews were excluded from analysis, but included in the discussion.

#### Population

The population included adolescents of both genders with AIS, diagnosed and managed between the ages of 10 to 18 years of age, with no restriction as to bone age (Risser sign). Curves of at least 11°, the borderline for the deformity to be diagnosed as scoliosis, measured on the A–P radiograph with the Cobb method, were eligible. All SRs addressing mild, moderate and/or severe AIS (11–24°, 25–44°, and 45°Cobb and greater, respectively) were included. Reviews on-early-onset (infantile or juvenile) scoliosis, as well as studies reporting on scoliosis secondary to other conditions, e.g. Duchenne dystrophy, cerebral palsy, spinal cord injury, neurofibromatosis were excluded.

#### Interventions

Eligible SRs addressed non-surgical interventions applied as a sole treatment or as combinations of different non-surgical interventions, and included:

braces of any type (both rigid and soft) and mode of application (any number of hours a day, or night-time),
any approach (s), or “school” of scoliosis-specific exercise treatment of AIS, regardless of the severity of the deformity, both as a single intervention, or as part of a group of different complex interventions, e.g. supplementing brace treatment (add-on treatment), chiropractic, manual therapy, electrical stimulation or general conditioning (usual) exercises.
SRs on any other non-surgical interventions were also considered.

Generalized and non-curve-specific exercises or other physiotherapeutic interventions administered to patients with AIS for other reasons, e.g. respiratory physiotherapy, spinal stabilization exercises or electrical stimulation due to low back pain or leg pain, were not the subject of this paper and were excluded. Studies relating to pre- or postoperative physiotherapeutic management of AIS patients, as well as to the natural history or observation (“watchful waiting”) as a form of therapy, were not included.

SRs on diagnostics, prognosis, economic analysis, or other research questions other than non-surgical interventions, were considered ineligible. These also applied for SRs or guidelines potentially including systematic reviews of evidence regarding screening for AIS. This subject matter has been reported separately [42].

#### Comparative interventions

The types of comparative interventions considered eligible were all non-surgical interventions as described below:

bracing, or scoliosis-specific exercises versus scoliosis-specific exercises plus other interventions, or different forms of these interventions (e.g. different modes of exercises, or different types of braces),
other forms of non-surgical interventions applied for scoliosis curve correction, e.g. chiropractic, manual therapy, electrical stimulation,
natural history or observation.

Natural history or observation were not eligible as a “tested” intervention, but were considered as comparators or comparative interventions (I and C in the PICO scheme, respectively).

#### Outcomes

All outcomes that addressed the effectiveness, as well as adverse effects of non-surgical interventions, both within the short and long term, were analyzed. These included both patient-centered (e.g. pain, quality of life, depression, sense of stigmatization) as well as surrogate, secondary or intermediate outcomes (e.g. curve progression, angle of trunk rotation, jaw deformity). The number of surgeries, or numbers needed to treat to avoid one surgery (need for surgery) as a criterion of failure of the non-surgical interventions were considered as well.

### Search methods for identification of papers

#### Electronic searches

The databases and other resources searched, as well as the order of searching, are detailed in Table 2. The search strategies, key words and limits used are detailed separately in Table S3. Searches in the general bibliographic databases were limited from 1980 or from the inception of a database (SportsDiscus –2001) to the latest possible current date. All SRs currently indexed in databases of SRs, databases separately indexing SRs and in guideline registries were considered. Time limits did not apply for websites of institutions, as these websites were assessed for current content. Electronic searches were last conducted between the 15 and 31 March, 2014.

**Table 2:** Databases searched and the order of searching.

**1. Databases of systematic reviews, databases with separate indexing of systematic reviews, guideline registries:** Cochrane Database of Systematic Reviews (CDSR); the Centre for Reviews and Dissemination databases (DARE, HTA, NHSEED); Joanna Briggs Institute: Database of Systematic Reviews and Implementation Reports, COnNECT+; Physiotherapy Evidence Database (PEDro); National Guideline Clearinghouse; Turning Research Into Practice (TRIP); Campbell Library
**2. Websites of institutions:** Scoliosis Research Society (SRS), Society for Spinal Orthopaedic and Rehabilitation Treatment (SOSORT), International Research Society for Spinal Deformities (IRSSD), Guidelines International Network (G-I-N), Scottish Intercollegiate Guideline Network (SIGN), National Institute for Clinical Excellence, UK (NICE), Agency for Healthcare Research and Quality (AHRQ), USA/Evidence-based Practice Centers: Evidence-based Reports, National Health and Medical Research Council, Australia (NHMRC)
**3. General bibliographic databases:** MEDLINE through PubMed, Web of Science: Science Citation Index – EXPANDED (SCI – EXPANDED), SportsDiscus through EBSCO

#### Hand searching

Hand searches of reference lists of included SRs, as well as in other relevant reviews, recommendations, guidelines, editorials, and other relevant papers, were conducted.

### Process of study selection

**Figure 1:**
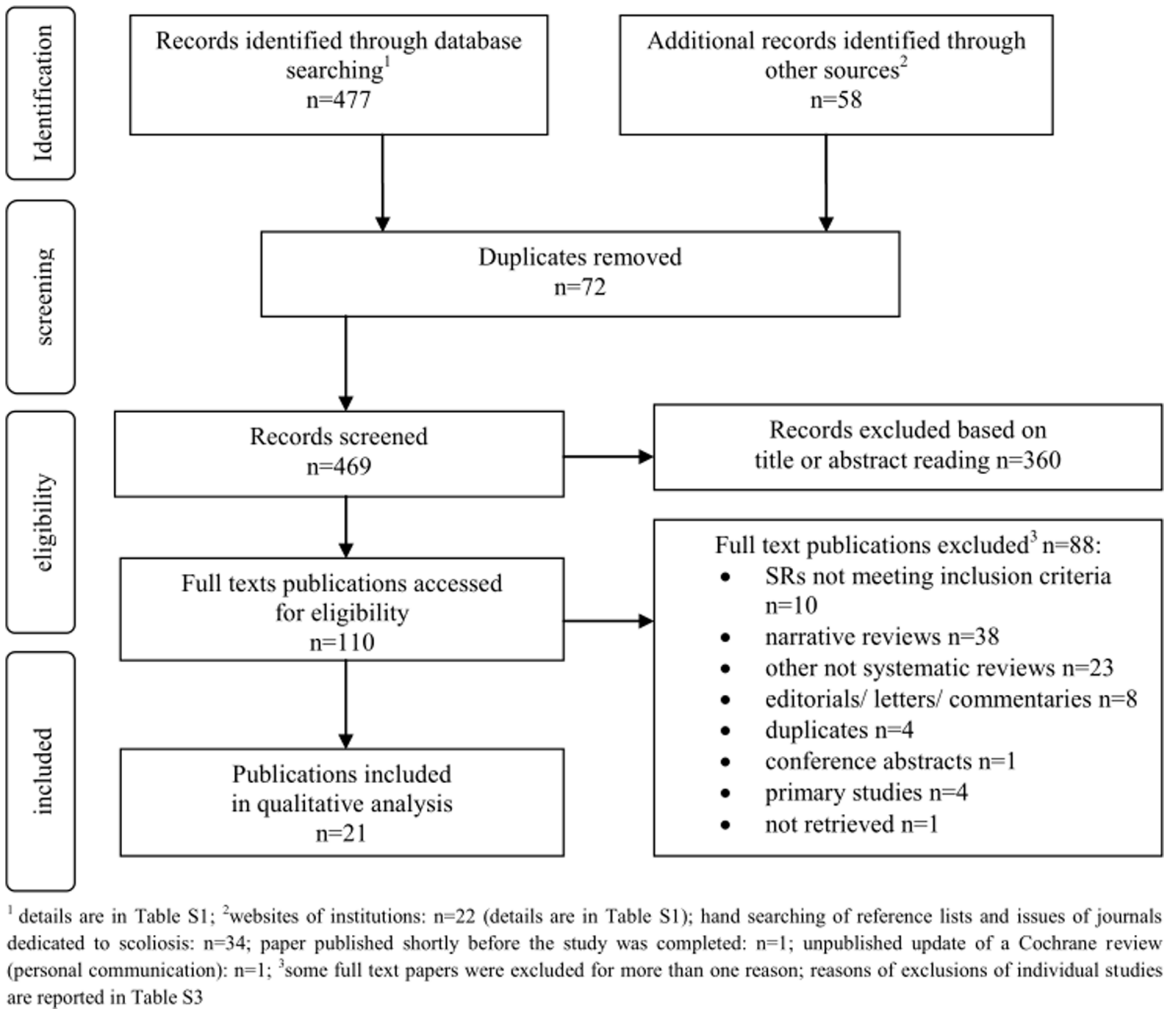
PRISMA flow diagram for the selection of included studies.

The initial search and screening of titles and abstracts to identify papers requiring closer scrutiny to assess their eligibility, was conducted by MP using the pre-defined criteria. This was conducted within databases and specialty websites, in the order presented in Table 2. The two authors then independently hand-searched the reference lists of all included reviews and proceeded to select the full papers potentially meeting all the inclusion criteria. Any disagreements were resolved through discussion. The PRISMA search flow diagram for the selection of included studies is shown in Figure 1.

As the aim of this overview was to analyze existing SRs, potential authors of unpublished SRs were not contacted neither were searches for gray literature, registered titles and review protocols conducted. The exception was one SR [43] for whom the first author was contacted with a request for supporting material mentioned in the paper which was not available from the publisher. An update of a Brace Cochrane review [33], co-authored by JB-S, being currently under review, was also considered.

### Process for the assessment of the methodological quality of included reviews

The “Assessment of Multiple Systematic Reviews”, (AMSTAR) risk of bias tool [44] was used to assess the **methodological quality** of included reviews. The AMSTAR tool is considered to be a valid and reliable instrument for assessing the methodological quality of reviews [45]. It comprises eleven items addressing criteria relating to the assessment of methodological rigor (Table 3). The items are scored “yes”, “no”, “cannot answer”, or “not applicable”. The maximum score is 11. Scores 0–4, 5–8, and 9–11 indicate low, moderate, and high quality reviews, respectively [46]. The appraisal was conducted independently by MP and JB-S. Exceptions were the Cochrane reviews [16], [33], that were included and coauthored by JB-S, when MP and a collaborator (IC) (invited for this purpose) performed the independent appraisals. Assessments were conducted using guidelines for scoring AMSTAR questions [44]–[46]. Disagreements were resolved by discussion.

**Table 3:** AMSTAR ratings for included reviews.

reference, year	AMSTAR questions^1^											total Yes	overall quality^2^
	1	2	3	4	5	6	7	8	9	10	11		
**exercise treatments:**													
[51], 2003 (1/3)^3^	N	CA	Y	N	N	Y	Y	Y	N	N	N	4	low
[52], 2008 (2/3)^3^	Y	CA	Y	N	N	Y	Y	Y	Y	N	N	6	moderate
[53], 2011 (3/3)^3^	Y	CA	N	N	N	Y	N^4^	N^5^	N	N	N	2	low
[54], 2012	N	CA^6^	N	N	N	Y	N	N	N	N	N	1	low
[33], 2012	Y	Y	Y	Y	Y	Y	Y	Y	Y	N	N	9	high
**manual therapy:**													
[6], 2008	N	CA	Y	N	N	Y	N	N^5^	N	N	N	2	low
[55], 2012	N	CA	Y^7^	N	N	Y	Y	N^8^	NA^9^	N	N	3	low
[56], 2013	N	Y	N	N	N	Y	Y^10^	Y	N	N	N	4	low
[7], 2013	N	Y	Y	N	N	Y	Y	Y	Y	N	Y	7	moderate
**bracing:**													
[25], 2007	N	N	Y	N	N	Y	N	N^5^	N	N^11^	N	2	low
[16], 2010	Y	Y	Y	Y	Y	Y	Y	Y	Y	N	N	9	high
[57], 2011	N	CA	N	N	N	Y	Y	Y	N	N	N	3	low
[43] ^12^, 2011	N	CA	CA^13^	N	N	Y	N	N^5^	Y	N	N	2^12^	low^12^
[36], 2012	N	CA	N	N	N	N	N	N	N	N	N	0	low
**different combinations of non-surgical interventions:**													
[58], 1991	N	Y	N	N	N	Y	Y	Y	N	N	N	4	low
[26], 1997	N	CA^14^	N	N^15^	Y	N^16^	N	N	Y	N	N	2	low
[37], 2005	N	Y	N^17^	N	N	Y	Y	Y	Y	N	N	5	moderate
[17], 2008	N	N	Y	N	N	N	N	N	N	N	N	1	low
**usual physical activity:**													
[59], 2009	N	Y	Y	Y	N	Y^18^	N	N^5^	N	N	N	4	low
**adverse effects:**													
[60], 2008	N	CA^19^	Y	N	N	Y	N	N	Y	N	N	3	low
[61], 2011	N	Y	Y	N	N	N^20^	N	N	N	N	N	2	low

Y – yes, N – no, CA – cannot answer, NA – not applicable

1 questions [44], [46]: “1. Was an a priori design provided?, 2. Was there duplicate study selection and data extraction?, 3. Was a comprehensive literature search performed?, 4. Was the status of publication (i.e. grey literature) used as an inclusion criterion?, 5. Was a list of studies (included and excluded) provided? 6. Were the characteristics of the included studies provided?, 7. Was the scientific quality of the included studies assessed and documented?, 8. Was the scientific quality of the included studies used appropriately in formulating conclusions?, 9. Were the methods used to combine the findings of studies appropriate?, 10. Was the likelihood of publication bias assessed?, 11. Were potential conflicts of interest included?”; ^2^scores of 0–4 indicate that the review is of low quality, 5–8 of moderate quality and 9–11 of high quality [44]; ^3^the 2003 review and two updates; the reviews differed in methodology, thus analysed, as explained in Tables 1 and S2; ^4^one new study added and its quality not assessed; ^5^limitations of the included studies are reported, but item 8 cannot be scored “Y” if item 7 is scored “N”; ^6^methods of data extraction not reported; ^7^minimal criteria for a “Y” met, though hand searching was limited to reference lists of two reviews published at least 2 years earlier; ^8^quality of the included RCT in AIS patients was assessed, but not used in formulating conclusions; ^9^the review addresses different conditions and different outcomes; ^10^we gave a “Y”, but only 4 out of 10 included studies were assessed with a methodological quality tool (PEDro scale); all 10 papers were classified with levels of evidence; ^11^could not score a “Y” if no formal assessment of heterogeneity performed (despite narrative descriptions of potential sources of heterogeneity between the studies); ^12^the paper states that “the details regarding the methodology can be found in the web appendix at www.aospine.org/esbj”; however, the website does not offer access to that appendix; when the publisher’s website was browsed the paper was not supplemented with an appendix from that site (http://www.thieme.com/index.php?page=shop.product_details&flypage=flypage.tpl&product_id=965&category_id=65&option=com_virtuemart&Itemid=53); similarly, the PubMed Central full text access is not supplemented with an appendix; finally, the first author was asked for this content, and, in reply, supplied the authors with a copy of the full text paper, and informed that the paper itself contains the information on methodology; thus, the AMSTAR analysis of the paper conducted without considering the appendix. ^13^ keywords and/or search strategy not available; ^14^three reviewers performed data extraction but it is not reported whether they worked independently; ^15^a thesis and unpublished report were included but they were not retrieved through searching for gray literature; ^16^some data from individual studies are provided, but description of participants and outcomes is lacking; ^17^the review meets the criterion of searching at least two sources, years and databases are reported, but there is no information on supplementary strategy (e.g. consulting current contents, textbooks, experts, reviewing reference lists [46];^ 18^scored a “Y” as included primary studies were characterised – the authors also included narrative reviews, which could not be characterised in terms of study participants or methodology;^ 19^unclear whether one or more people independently conducted study search and selection process; ^20^this study was an extremely poor study which was very difficult to make sense of

**The level of evidence** from each included SRs was assessed, considering the types of primary (and, in individual reviews, also secondary) studies included, using the Oxford Centre for Evidence Based Medicine (OCEBM) [47], [48] and the Joanna Briggs Institute (JBI) [49], [50] classifications.

#### Data extraction and management

The data was independently extracted by MP and JB-S, using predefined data extraction forms (Tables 4 and 5). Discrepancies were resolved through discussion.

**Table 4:** Content (PICO) characteristics of included reviews.

title, reference, year	Participants/condition(s)	Intervention(s)	Comparator(s)	Outcome measure(s)
**EXERCISE TREATMENTS:**				
*Physical exercises as a treatment for adolescent idiopathic scoliosis. A systematic review.* 2003; [51]; (SR 1 of 3)^1^	patients with AIS, Risser sign <5 (skeletally immature)	any type of treatment with SSEs^3^, but not mixed with other treatments	any type, or no treatment	Cobb angle/curve progression
*Exercises reduce the progression rate of adolescent idiopathic scoliosis: results of a comprehensive systematic review of the literature*. 2008; [52]; (SR 2 of 3)^1^	patients with AIS, Risser sign <5 (skeletally immature)	any type of treatment with SSEs^3^, but not mixed with other treatments	any type, or no treatment	Cobb angle/curve progression
*Physical exercises in the treatment of adolescent idiopathic scoliosis: an updated systematic review.* 2011; [53]; (SR 3 of 3)^1^	patients with AIS, Risser sign <5 (skeletally immature)	any type of treatment with SSEs^3^, but not mixed with other treatments	any type, or no treatment	Cobb angle/curve progression
*Efficacy of exercise therapy for the treatment of adolescent idiopathic scoliosis: a review of the literature.* 2012; [54]	patients with AIS	exercise therapy (as a sole therapy)	different, reported individually for the included controlled studies	inclusion criterion: “minimum of one defined outcome measure; outcome measures reported individually for the included studies
*Exercises for adolescent idiopathic scoliosis.* 2012; [33]; Cochrane review	AIS, age from 10 years to skeletal maturity (Risser sign or ages between 15–17 years in girls or 16–19 years in boys)	all types of SSEs^2^;	no treatment, different types of SSEs, usual physiotherapy, doses and schedules of exercises, other non-surgical treatments	“a review of the effect of the exercise on a radiological observation rather than a clinical syndrome”; primary outcomes: curve progression, cosmetic issues, QoL and disability, back pain, psychological issues; secondary outcomes: adverse effects;
**MANUAL THERAPY:**				
*Manual therapy as a conservative treatment for adolescent idiopathic scoliosis.* 2008; [6]	patients with AIS	“manipulative therapeutic methods”: osteopathic, chiropractic, massage techniques	none of the included studies had a control group/comparator intervention	curve progression (Cobb angle)
*The use of spinal manipulative therapy for pediatric health conditions: a systematic review of the literature.* 2012 [55]	IS (among various clinical conditions affecting children); age ≤18 years	“manual, high-velocity low-amplitude thrusting spinal manipulations”	standard medical care or sham manipulation	“studies using some type of outcome measure for determining the effect of chiropractic care”; the AIS study: Cobb angle, QoL
*Myofascial release as a treatment for orthopaedic conditions: a systematic review.* 2013; [56]	IS among other orthopaedic conditions	indirect and passive myofascial release (MFR)	not applicable – one case study included	OMs not defined as inclusion criteria for the SR; OMs from the included IS case study: pain, pulmonary function, QoL, range of motion
*Osteopathic manipulative treatment for pediatric conditions: a systematic review.* 2013; [7]	adolescents with IS (IS among other paediatric conditions)	OMT^3^ (performed by osteopaths) as a sole treatment; studies on chiropractic manipulations excluded; the OMT technique used in the AIS study described in detail	any type of controls admissible; control in the AIS study – no treatment	any outcome measures in RCTs investigating the effect of OMT (e.g. hospital stay, spine flexibility, FEV_1_– reported for individual studies), also in conjunction with other treatment, on paediatric conditions, included;
**BRACING:**				
*Surgical rates after observation and bracing for adolescent idiopathic scoliosis: an evidence-based review.* 2007; [25]	patients with AIS, and no other conditions responsible for curvature: 20–45°Cobb, Riser 0–2, age <15 years, follow-up to skeletal maturity	bracing as a sole treatment (e.g. without physical therapy): TLSOs, bending braces, but not Milwaukee, SpinalCor, Triac;	observation	surgery; recommended surgery; curve progression >50°
*Braces for idiopathic scoliosis in adolescents.* 2010; [16]; Cochrane review	patients with AIS, aged from 10 years to skeletal maturity	all types of braces, “worn for a specific number of hours a day for a specific number of years”;	any control interventions and comparisons	primary: pulmonary disorders, disability, back pain, QOL, psychological and cosmetic issues; secondary: cob angle and progression >5° before, at the end of bone maturity, in adulthood; adverse effects
*Effectiveness and outcomes of brace treatment: A systematic review.* 2011; [57]	patients with AIS	bracing (any type)	observation; exercises; LESS; casting; surgery	curve progression; surgery; pulmonary function; QoL; “psychological state”
*Efficacy of bracing versus observation in the treatment of idiopathic scoliosis.* 2011; [43]	patients with AIS	bracing (any type/method);	observation	surgery rates, failure rates, QoL, curve angle changes
*Bracing in adolescent idiopathic scoliosis, surrogate outcomes, and the number needed to treat.* 2012; [36]	patients with AIS	bracing	natural history	6° curve progression – surrogate outcome vs need for surgery in NNTs; efficacy of different braces
**DIFFERENT COMBINATIONS OF NON-SURGICAL INTERVENTIONS:**				
*Effectiveness of non-surgical treatment for idiopathic scoliosis. Overview of available evidence.* 1991; [58]	juveniles and adolescents with IS	natural history; non-surgical treatment: LESS, bracing (Milwaukee and/or Boston or Wilmington, full-time or part-time), posture training device, exercises (not specified)	Non-treated controls in one included study; other studies non-controlled	progression (>5°Cobb); failure of treatment (>45°Cobb or surgery)
*A meta-analysis of the efficacy of non-operative treatments for idiopathic scoliosis.* 1997; [26]	juveniles and adolescents with IS	bracing: Milwaukee, TLSO: 8, 16 and 23 hours/day; Charleston; LESS; observation	only one included study had a control group (bracing vs no treatment)	type of treatment, level of maturity (Risser sign), criterion to determine progression (or failure of treatment): 3°, 5°, 6° or 10°Cobb, duration of brace wear
*Effect of bracing and other conservative interventions in the treatment of idiopathic scoliosis in adolescents: a systematic review of clinical trials.* 2005; [37]	IS, patients aged <18 years	bracing: Boston, Milwakee, Chêneau, Charlston, TLSO, underarm plastic, 3-valve orthosis; different types and wearing times; exercise+brace; exercise program; Side-Shift; SSE+ES; LESS; night-time ES; behaviourally posture-oriented training; traction; fixed traction at night	different control interventions, or combinations of interventions, individual trials included	measures of effectiveness, depending of the study included: Cobb angle, rotation component, loads on instrumented pads (Milwakee brace, throat mold design); surgery rate (number of surgeries undertaken); success rate
*The treatment of adolescent idiopathic scoliosis (AIS) according to present evidence. A systematic review.* 2008; [17]	patients with AIS	physiotherapy, rehabilitation, bracing, surgery^4^	observation	not specified
**USUAL PHYSICAL ACTIVITY:**				
*Is physical activity contraindicated for individuals with scoliosis? A systematic literature review.* 2009 [59]	people with all types of scoliosis	usual physical and sports activities; treatment options: observation, bracing (type of brace not reported as a criterion), surgery^4^; therapeutic exercise(s): exclusion criterion	physical activity of healthy subjects – in 2 included case-controlled studies; other studies uncontrolled	any measures related to appropriateness of physical/sports activity
**ADVERSE EFFECTS:**				
*Low-bone mineral status in adolescent idiopathic scoliosis.* 2008; [60]	adolescents with IS	the association between brace wear and bone mineral density were evaluated	different, reported individually for the included controlled studies	low bone mass in idiopathic scoliosis
*Scoliosis and dental occlusion: a review of the literature.* 2011; [61]	adolescents with malocclusion and scoliosis	the paper concentrated on prevalence and coincidence of malocclusion and scoliosis, but also on effects of bracing intervention (bracing) on malocclusion^5^	non-scoliosis subjects in the individual controlled primary studies (reported separately), but uncontrolled studies also included	any descriptions of outcomes of interest: primary: “incidence and description of malocclusion of people with scoliosis” secondary: “clinical consequences associated with treatments of malocclusion or scoliosis”

AIS – adolescent idiopathic scoliosis; FEV_1_– forced expiratory volume in 1 second; NA – not addressed; NNT - Number Needed to Treat; OM – outcome measure; QoL – quality of life; SIR – “scoliosis inpatient rehabilitation”; SR – systematic review;

1 series of updates, analysed in concert or separately, depending on how the authors addressed individual study characteristics (also explained in Table S1);

2 SSE – scoliosis specific exercise: “curve-specific movements performed with a therapeutic aim of reducing the deformity” [33];

3 OMT - Osteopathic manipulative treatment, defined in [7] as “the therapeutic application of manually guided forces by an osteopathic physician to improve physiologic function and/or support homeostasis that has been altered by somatic dysfunction”;

4 recommendations related to surgically treated patients not analysed here;

5 malloclusion – “Imperfect positioning of the teeth when the jaws are closed” [http://www.oxforddictionaries.com/definition/english/malocclusion].

**Table 5:** Methodological characteristics of included systematic reviews.

title, reference, year	searching	included studies		method of synthesis	remarks
		number, type	quality assessment		
**EXERCISE TREATMENTS:**					
*Physical exercises as a treatment for adolescent idiopathic scoliosis. A systematic review.* 2003; [51]; (SR 1 of 3)^1^	MEDLINE, EMBASE, CINAHL, Cochrane Library (no particular database reported), PEDro	n = 11∶5 uncontrolled before-after; 6 controlled (2 with a historical control)	**11 quality criteria assessed**; not referred to any validated scoring tool	study characteristic in tables and figures; individual studies described narratively	
*Exercises reduce the progression rate of adolescent idiopathic scoliosis: results of a comprehensive systematic review of the literature*. 2008; [52]; (SR 2 of 3)^1^	MEDLINE, EMBASE, CINAHL, Cochrane Library (no particular database reported), PEDro; hand searching	n = 8 new: 1 RCT, 3 other controlled, 4 uncontrolled	**11 quality criteria assessed**; not referred to any validated scoring tool	study characteristic in tables and figures; individual studies described narratively	
*Physical exercises in the treatment of adolescent idiopathic scoliosis: an updated systematic review.* 2011; [53; (SR 3 of 3)^1^	MEDLINE, EMBASE, CINAHL, Cochrane Library (no particular database reported), PEDro; hand searching	n = 1 new PC	characteristic of individual studies	descriptive characteristics; studies not assessed for heterogeneity	study design not an inclusion criterion
*Efficacy of exercise therapy for the treatment of adolescent idiopathic scoliosis: a review of the literature.* 2012; [54]	PubMed; MEDLINE (separately from PubMed, with different results reported); EMBASE; Cochrane Library	n = 12∶9 PC (3 controlled); 2 RC; 1 CS	LoE for each study defined; characteristic of individual studies	characteristic of individual studies in tables and narratively	
*Exercises for adolescent idiopathic scoliosis.* 2012; [33]; Cochrane review	CENTRAL, MEDLINE, EMBASE, CINAHL, SportsDiscus, PsycInfo, PEDro; reference lists; trial registries; grey literature; contacts with authors and investigators	n = 2∶1 RCT; 1PC	**quality scoring tools:** Cochrane risk of bias tool for RCTs and CCTs; NOS scale for PCs;clinical relevance: 5 standard questions;characteristic of individual studies	dichotomous outcomes: RR for each trial; continuous outcomes: MD and SMD; two studies included and assessed for clinical heterogeneity, therefore no meta-analysis performed; overall quality of evidence for each outcome: **adapted GRADE approach**	
**MANUAL THERAPY:**					
*Manual therapy as a conservative treatment for adolescent idiopathic scoliosis.* 2008; [6]	MEDLINE, EMBASE, CINAHL, Cochrane Library, PEDro, ICL, Osteomed, Osteopathic Research Web, NCCAM	n = 3∶1 C; 1 CS; 1 pilot/feasibility study; additionally, 3 CS discussed	characteristics of individual studies; clinical relevance of studies assessed using five questions recommended by Cochrane Back Review Group	descriptive characteristics of included studies	types of manipulation techniques not defined/diversified; studies described narratively; no formal quality assessment and data synthesis;
*The use of spinal manipulative therapy for pediatric health conditions: a systematic review of the literature.* 2012 [55]	ICL, PubMed; reference lists of previously published reviews	n = 1 pilot RCT on AIS (17 studies regarding other conditions)	10 item **quality assessment tool** developed by Sackett, maximal score 50 points; characteristics of individual studies	characteristics of individual studies in a table and narratively	quality score not used to draw conclusions regarding AIS; conclusions based on 1 small feasibility pilot study
*Myofascial release as a treatment for orthopaedic conditions: a systematic review.* 2013; [56]	MEDLINE, CINAHL, Cochrane Library, PEDro, Academic Search Premier	n = 1 CS on AIS (4 RCTs and 5CSs regarding other conditions)	**quality scoring tool:** PEDro scale for RCTs (4 studies assessed); LoE (CEBM Oxford) for each of 10 studies defined; characteristic of individual studies	characteristic of individual studies in a table and narratively	the paper is in part a report of an SR of experimental studies (RCTs, as we conclude from the LoEs stated and quality appraisal tool used), and in part a narrative analysis of case studies
*Osteopathic manipulative treatment for pediatric conditions: a systematic review.* 2013; [7]	AMED, CINAHL, Embase, Medline, OSTMED.DR, PsycINFO, Cochrane Library, PEDro, ISI Web of Knowledge, Osteopathic Research Web, Rehabdata; reference lists of retrieved studies and key SRs of OMT	n = 1 RCT on OMT in mild AIS (16 RCTs regarding other conditions)	**Cochrane risk of bias tool**; characteristic of individual studies	effect sizes for the effect of OMT on any type of OM calculated by using Cohen’s d formulas; characteristic, quality and limitations of included RCTs discussed both in concert, and separately for each study	
**BRACING:**					
*Surgical rates after observation and bracing for adolescent idiopathic scoliosis: an evidence-based review.* 2007; [25]	MEDLINE, Web of Science, CENTRAL, Clinical Evidence, reference lists	bracing n = 15∶4 R comparison studies, 8 R CS, 2 P CS, 1 P/R CS; observation n = 3 R comparison studies	rating system (LoE); characteristics of individual studies	**pooled prevalence estimates** of surgery in untreated and brace treated patients; pooled prevalence for surgery by type of brace, curve type, skeletal maturity and dose (full time vs part time wear)	search limited to English language; quality assessment not conducted; one reviewer selected studies and extracted data; studies not tested for heterogeneity
*Braces for idiopathic scoliosis in adolescents.* 2010; [16]; Cochrane review	CENTRAL, MEDLINE, CINAHL, up to July 2008; reference lists; trial registries; grey literature; contacts with authors and investigators	n = 2∶1 RCT; 1 multicentre international PC	**Quality scoring tools:** Cochrane risk of bias tool for RCTs and CCTs; NOS for PCs; characteristic of individual studies	Meta-analysis not performed as only 1 RCT and 1 PC included; overall quality of evidence for each outcome assessed with an **adapted GRADE approach**	
*Effectiveness and outcomes of brace treatment: A systematic review.* 2011; [57]	PubMed	n = 20∶2 CCT; 18 C–C	**11 criteria of methodological quality** (Cochrane Back Review Group)	characteristics of individual studies; CCTs and C–C assessed with the same criteria, and analysed collectively; LoE ratings provided, but not used	low quality of reporting: different papers included (selected) and different ones analysed in “discussion” section
*Efficacy of bracing versus observation in the treatment of idiopathic scoliosis.* 2011; [43]	PubMed, Cochrane Collaboration database (no particular database reported), NGC; references of “key articles”	n = 8∶5 PC; 3 RC comparison studies	characteristic of individual studies, addressing QoL, curve angle and surgery rates; rating system: SoE	pooling of data impossible due to heterogeneity; pooled surgical rates and between groups risk differences calculated from study-level data; treatment effects calculated by subtracting change scores; descriptive characteristics of individual studies;	Comparison (analytic design) cohort studies included; RCT an inclusion criterion – no RCTs found; findings based on “the highest” obtainable (comparison studies), but still “very low to low” level of evidence
*Bracing in adolescent idiopathic scoliosis, surrogate outcomes, and the number needed to treat.* 2012; [36]	Cochrane Database (not stated, which particular one), PubMed Clinical Queries	two separate searches: question 1: bracing efficacy: n = 6 (3SRs, 2 P (1 RCT), 1 R CS); question 2: studies comparing different braces: n = 11 (1 RCT, 1 CCT, 2 SRs, 9 R comparison studies)	included studies of various types discussed shortly in a narrative; LoEs reported as quality of evidence analysis	included studies characterised individually in a narrative, including methodological assessment of some of them; one cohort study analysed in detail, with NNTs calculated and discussed	review of studies of various types, including SRs; one CS analysed in detail; the manuscript consists partly of the report of SR of studies of various types, and partly of an educational presentation of the issue of NNTs and surrogate outcomes
**DIFFERENT COMBINATIONS OF NON-SURGICAL INTERVENTIONS**					
*Effectiveness of non-surgical treatment for idiopathic scoliosis. Overview of available evidence.* 1991; [58]	MEDLINE, 1975–1987; reference lists	natural history n = 17∶4 PC, 3 RC; bracing n = 10∶7 RC, 1 PC, 2 unclear data; bracing+exercise: n = 1 PC; LESS: n = 5∶3 PC, 1 RC, 1 unclear P	characteristics of individual studies	**pooled proportions of data**; descriptive characteristics of included studies;	searching incomprehensive; lack of quality appraisal of the included studies; only uncontrolled studies included; data from studies on juvenile and adolescent IS mixed
*A meta-analysis of the efficacy of non-operative treatments for idiopathic scoliosis.* 1997; [26]	Campbell’s Operative Pediatric Orthopedics, 2^nd^ ed., 1995; 2 publications added by authors	n = 19∶18P and 1R: 13 on bracing; 6 on LESS; 1 on observation; 18 published (1 thesis); 1 unpublished	characteristics of individual studies	number of failures of treatment and mean proportion of success determined for each study; then data combined in **meta-analysis**; Q test to assess homogeneity; other tests to determine the contribution of variables	one data source searched, details of searching lacking; limited data on included studies and their quality; results not adjusted for compliance to brace wear; potential differences in LESS technique not considered
*Effect of bracing and other conservative interventions in the treatment of idiopathic scoliosis in adolescents: a systematic review of clinical trials.*2005; [37]	Cochrane, CINAHL, PubMed, PEDro	n = 13∶3 RCTs: 2 on bracing+exercises; 1 on traction; 10 CCTs: 7 on different rigid braces; 1 on side-shift therapy; 1 on behaviourally posture-oriented training; 1 on LESS	**quality scoring tool:** Delphi list, quality scores; rating system (LoE); characteristics of individual studies	quality of individual studies assessed; where possible, relative risks calculated; studies assessed for homogeneity	some AMSTAR items scored “N”, but criteria partially met (Table 2); many interventions and outcomes considered, with few weak quality primary studies available
*The treatment of adolescent idiopathic scoliosis (AIS) according to present evidence. A systematic review.* 2008; [17] 2	PubMed, MEDLINE, Web of Science, EMBASE, DH-DATA, Allied and Complementary Medicine, British Nursing Index, CENTRAL, CINAHL, PsycInfo; reference lists	physiotherapy: n = 6 “level III studies with control groups and cohort studies”, n = 2 SRs; bracing: n = 2 “level II studies” –1 P controlled multicenter, 1 P controlled long-term follow-up; 26 R or uncontrolled series, 1 meta-analysis	discussion of chosen papers, general impression of the LoEs	general assumption of the LoEs (Oxford CEBP) from the included studies; descriptive characteristics of some of the included studies	search strategy reported, but only keywords provided; quality appraisal: only levels of evidence mentioned, but appraisal/scale/scoring not addressed or reported; the paper is in part a report from an SR of evidence, and in part a narrative review
**USUAL PHYSICAL ACTIVITY:**					
*Is physical activity contraindicated for individuals with scoliosis? A systematic literature review.* 2009; [59]	PubMed, CINAHL, ICL, NGC, reference lists; websites of organisations	n = 11∶3 C–C; 1 CS; 1 survey; 6 narrative reviews	rating system (LoE) used (but reported as quality assessment); characteristic of individual studies	descriptive characteristics of included studies	study type not an inclusion criterion −6 out of 11 papers included were narrative reviews; opinion-based recommendations rather than evidence-synthesis
**ADVERSE EVENTS:**					
*Low bone mineral status in adolescent idiopathic scoliosis.* 2008; [60]	MEDLINE; EMBASE; CINAHL; All EBM Reviews (Cochrane DSR, CCTR, DARE, ACP Journal Club); reference lists	5 brace studies of different characteristic (no details on design provided)	no formal quality assessment due to “wide variations”across studies; characteristics of individual studies	pooling of data not performed due to heterogeneity; descriptive characteristics of included studies	lack of clear information on the types of included studies; no quality assessment performed
*Scoliosis and dental occlusion: a review of the literature.* 2011; [61]	MEDLINE, EMBASE, Cochrane Oral Health Group Trial Register, handsearching: orthodontic journals, reference lists	n = 2 referenced, 1 (CCT ?) regarding the effect of Milwaukee brace on dentofacial growth analysed narratively	design, number and age of participants, and type of control group	descriptive characteristics of the included study	low quality of reporting: e.g. errors in referencing, “discussion” and “conclusions” sections do not address results of the evidence synthesis

B – bracing; Ex – exercises; O – observation; Surgery; NR – not reported; P –prospective study; R – retrospective study; C - cohort study; CS – case series; LESS – lateral electrical stimulation; ES – electrical stimulation; RCT – randomised controlled trial; CCT – controlled clinical trial; C–C – case-control study; NCCAM – National Centre for Complementary and Alternative Medicine; ICL – the Index to Chiropractic Literature; NGC – National Guideline Clearinghouse; QoL – quality of life; CENTRAL – the Cochrane Central Register of Controlled Trials; NOS scale – the Nottingham-Ottawa scale; LoE – level of evidence; SoE – strength of evidence; SR – systematic review; SSE - scoliosis-specific exercises; ATR – angle of trunk rotation; RR – risk ratio; MD – mean difference; SMD – standardised mean difference; OMT – osteopathic manipulative treatment;

1 series of updates, analysed in concert or separately, depending on how the authors addressed individual study characteristics (also explained in Table S1);

2 the review has a section on surgical treatment, not reported here.

#### Data synthesis

All the data extracted from the SRs was grouped by intervention and adverse effects. Narrative summaries of the review questions as well as eligibility criteria, populations studied, outcome measures and findings were then listed separately for individual reviews, and presented in Table 4.

The methodological characteristics of the included SRs – sources searched, selection criteria, methods of quality assessment of included studies, methods of data extraction and synthesis, and methodological limitations of the SRs were reported in the same order and can be seen in Table 5.

## Results

### Search

After removal of duplicates, 469 titles or titles and abstracts were screened for inclusion, 360 titles and/or abstracts were excluded, 110 full text papers were analyzed and 21 SRs were included for data synthesis and quality analysis (Figure 1). Four guideline documents addressing the subject matter were found, but none of them met the inclusion criteria.

The SRs that were included are listed in Table S1 with reasons for inclusion in cases where this was not clear. Excluded papers are listed, with the research designs classified, and the rationale for exclusion explained in Table S2.

Eighteen SRs addressed the effectiveness of non-surgical interventions: five SRs addressed SSE methods [33], [51]–[54], four evaluated manual therapies [6], [7], [55], [56] and five addressed bracing [16], [24], [36], [43], [57]. Four SRs compared the effectiveness of different interventions: bracing, therapeutic SSEs, lateral electrical surface stimulation (LESS), observation and/or surgery, or else their combinations (e.g. bracing plus exercises) [17], [26], [37], [58]. One review evaluated usual physical activity [59]. Two SRs addressed side effects: low bone status [60] and malocclusion [61] in braced patients. Overall the reviews addressed numerous, patient-centered and surrogate short and long-term outcomes. The types of interventions examined, types of participants, outcomes and authors’ conclusions are presented in detail in Table 4.

### Other reviews found

#### Complementary and alternative medical interventions (CAM)

Whilst primary studies of non-surgical CAM interventions have been reported in the literature (acupuncture, herbal treatment, or Pilates [8]) no SRs (secondary analyses) addressing any of these approaches could be found.

#### Overviews of reviews

Two overviews of reviews evaluating non-surgical interventions for AIS were found: a narrative review [62], a systematic overview of systematic reviews [63] which included one eligible SR [6] among other SRs regarding manipulative therapies in various pediatric conditions. This SR was also found through the search process and was included in the analysis.

### Methodological quality of included reviews

#### AMSTAR scores

Analysis with the AMSTAR tool revealed that the large majority of included reviews, 16 out of 21 included reviews were of low methodological quality, with scores ranging from 0 [36] through 1 [17], [54], 2 [6], [25], [26], [43], [53], [61] and 3 [55], [57], [60] to 4 points [51], [56], [58], [59]. Three moderate quality SRs scored 5 [37], 6 [52] and 7 [7]. Two SRs (Cochrane reviews) [16], [33] were of high methodological quality, and scored 9. Table 3 provides details of the AMSTAR quality assessment for each included SR, with explanations regarding the scoring decisions.

#### Narrative content analyses of methodological issues in included reviews

The SRs differed with regards to the sources of data as well as the databases searched. Three SRs were based on searches of one database [57], [58] or a textbook (?!) that was used by Rowe et al. [26] and seven SRs [6], [36], [37], [51], [54], [56], [57] were based exclusively on electronic searches. The only SRs where authors and investigators were contacted as a method of retrieving data were the Cochrane reviews [16], [33].

Eighteen SRs included only AIS patients. Two of these reviews [16], [33] were Cochrane reviews of randomized controlled trials (RCTs) and other prospective controlled studies. One SR [37] analyzed RCTs and nonrandomized controlled trials. Fourteen SRs included a diverse mix of primary studies that included both experimental and observational designs. One SR [59] considered both primary studies of various designs and narrative reviews. The remaining three SRs [7], [55], [56] considered an AIS population that was included within other pediatric conditions: one review was a SR [7] of RCTs and included one RCT on AIS, one SR included different controlled studies amongst them a single pilot RCT on AIS [55], and one [56] included a case study on AIS.

Seven SRs [7], [16], [33], [37], [55]–[57] included the analyses of the methodological quality of individual studies, using validated scoring tools. In six SRs hierarchies of levels of evidence (LoE) [17], [25], [54], [59] or strengths of evidence (SoE) [36], [43] were used as a way (or rather instead of) assessing the methodological quality of primary studies. In one SRs [37] the LoE hierarchy assessment supplemented the quality appraisal of the included reports. One SR [56] provided LoEs for all the included studies, but a quality assessment for the RCTs only.

One of the SRs [26] included a meta-analysis, one SR comprised a pooled prevalence estimates [25], whilst in another SR the authors had performed a pooled proportions of data [58]. The remaining papers provided descriptive analyses of individual studies.

A detailed narrative analysis of the methodological issues within the included SRs can be seen in Table 5.

### Levels of evidence, findings and conclusions

The evidence from included reviews is summarized in Table 6, according to each non-surgical intervention and in the order of descending levels of evidence.

**Table 6:** Evidence from systematic reviews on non-surgical interventions in AIS, in the order of descending levels of evidence.

reference, year	findings/conclusions	level of evidence [OCEBM/JBI]^1^	AMSTAR score^2^/overall quality
**EXERCISE TREATMENTS:**			
*Exercises for adolescent idiopathic scoliosis.* 2012; [33]; Cochrane review	“due to a lack of high quality RCTs in this area, there is no evidence for or against exercises, so hardly any recommendations can be given”; “no major risks of the intervention have been reported (…), and no side effects were cited in the considered studies”	1/1a	9/high^3^
*Exercises reduce the progression rate of adolescent idiopathic scoliosis: results of a comprehensive systematic review of the literature*. 2008; [52]; (SR 2 of 3)^4^	“Exercises can be recommended according to level-1b [OCEBM]^4^ evidence with the aim of reducing scoliosis progression”; “it is impossible to state anything regarding the kind of exercises. [or].kind of auto-correction to be performed”	3/1b	5/moderate
*Physical exercises as a treatment for adolescent idiopathic scoliosis. A systematic review.* 2003; [51]; (SR 1 of 3)^4^	“the efficacy of physical exercises in the treatment of AIS to reduce progression of the curve remains to be demonstrated”; “their utility to reduce specific impairments and disabilities [breathing function, strength, postural balance] (…) cannot be neglected”	3/1b	4/low
*Efficacy of exercise therapy for the treatment of adolescent idiopathic scoliosis: a review of the literature.* 2012; [54]	methodological flaws of included studies; majority of evidence from studies performed in centres specialising in exercise treatment; “the current literature review failed to find robust evidence in support of exercise therapy in the treatment of AIS”	3/1b	1/low
*Physical exercises in the treatment of adolescent idiopathic scoliosis: an updated systematic review.* 2011; [53]; (SR 3 of 3)^4^	conclusions from the preceding review [52] maintained	4^5^/3b	1/low
**MANUAL THERAPY:**			
*Osteopathic manipulative treatment for pediatric conditions: a systematic review.* 2013; [7]	findings from the AIS RCT: no evidence to support OMT as an effective treatment of mild AIS; the study assessed as high quality RCT; “more robust RCTs are needed (…). Until such data are available, OMT cannot be regarded as effective therapy for paediatric conditions, and osteopaths should not claim otherwise”	1/1a	7/moderate
*The use of spinal manipulative therapy for pediatric health conditions: a systematic review of the literature.* 2012; [55]	no conclusions regarding treatment effectiveness formulated both in the SR, and – as reported in the SR – in the included feasibility study	2^6^/1a	3/low
*Myofascial release as a treatment for orthopaedic conditions: a systematic review.* 2013; [56]	findings from the IS case study: at the end of treatment the patient showed improvement in the outcomes measured; the study considered “lower quality in design”, but the results are “very promising and give the foundations for future RCTs”	4^7^/1b	4/low
*Manual therapy as a conservative treatment for adolescent idiopathic scoliosis.* 2008; [6]	no evidence to draw any conclusions, complete lack of studies of acceptable quality, in opposition to brace and exercise therapy studies; urgent need for research	4/3b	2/low
**BRACING:**			
*Braces for idiopathic scoliosis in adolescents.* 2010; [16]; Cochrane review	very low quality of evidence in favour of bracing in terms of curve progression; low evidence in favour of hard bracing vs elastic bracing; serious side effects not documented in the included studies	1/1a	9/high^3^
*Efficacy of bracing versus observation in the treatment of idiopathic scoliosis.* 2011; [43]	“Findings with respect to surgical rates, quality of life, and change in curve angle demonstrate either no significant differences or inconsistent findings favouring one treatment or the other [bracing or observation].”; “If bracing does not cause a positive treatment effect, then its rejection will lead to significant savings for healthcare providers and purchasers.”	3/2a	2/low^8^
*Effectiveness and outcomes of brace treatment: A systematic review.* 2011; [57]	limited evidence suggests that bracing can prevent curve progression (compared to observation), may not negatively influence quality of life, may be more effective than ES; it is not known if bracing is better than Side-Shift therapy and casting; bracing and surgery cannot be compared due to differences in study groups	3/2b	3/low
*Surgical rates after observation and bracing for adolescent idiopathic scoliosis: an evidence -based review.* 2007; [25]	“Inconclusive and inconsistent evidence concerning the risk of surgery in AIS”; the review “did not demonstrate any advantage to bracing over surgery in terms of surgical rates”	4/3b	2/low
*Bracing in adolescent idiopathic scoliosis, surrogate outcomes, and the number needed to treat.* 2012; [36]	The NNT is about 9 braced patients for 1 surgery, but about 4 for patients highly compliant; however the NNTs are derived from nonrandomised cohorts and should be treated with caution; bracing may reduce the need for surgery; there is no evidence for one brace over another, but rigid bracing seems better than soft braces (SpineCor)	CA/1b^9^	0/low
**DIFFERENT COMBINATIONS OF NON-SURGICAL INTERVENTIONS:**			
*Effect of bracing and other conservative interventions in the treatment of idiopathic scoliosis in adolescents: a systematic review of clinical trials.* 2005; [37]	“Effectiveness of bracing and exercises is promising but not yet established’; limited evidence for the effectiveness of braces vs no treatment and vs electrical stimulation (ES); bracing, exercises or ES as add-on treatment – additional effect cannot be justified; no difference for: ES vs no treatment, bracing vs exercises, different types of bracing	1/1b	5/moderate
*The treatment of adolescent idiopathic scoliosis (AIS) according to present evidence. A systematic review.* 2008; [17]	“weak evidence (level III and IV) to support outpatient physiotherapy”; “evidence for the application of scoliosis inpatient rehabilitation”; various types, working mechanisms of braces, outcome measures in analysed studies, and “no definite or collective meaning for brace as such”, prevent from drawing conclusions on effectiveness of bracing	CA/2b^10^	1/low
*Effectiveness of non-surgical treatment for idiopathic scoliosis. Overview of available evidence.* 1991; [58]	early brace treatment may prevent severe progression and surgery in considerable proportion of patients, but controlled studies are needed	3/2b	4/low
*A meta-analysis of the efficacy of non-operative treatments for idiopathic scoliosis.* 1997; [26]	bracing more effective than LESS or observation; LESS not more effective than observation; bracing for 23 hours/day effective; skeletal maturity and criterion for failure significantly influence outcomes	CA (4?)/CA^11^	2/low
**USUAL PHYSICAL ACTIVITY:**			
*Is physical activity contraindicated for individuals with scoliosis? A systematic literature review.2009; [59]*	recommendations: observed and brace treated patients encouraged to participate in sports and physical activities (with or without braces on) – grade D of recommendations (OCEBM)^12^	4/4a^10^	4/low
**ADVERSE EFFECTS:**			
*Low bone mineral status in adolescent idiopathic scoliosis.* 2008; [60]	included studies do not support the presumption that bracing for AIS results in permanent loss of mineral bone mass; however, study findings are of limited value due to lack of data on compliance	CA/CA^13^	3/low
*Scoliosis and dental occlusion: a review of the literature.* 2011; [61]	the only relevant information from the review is that Milwaukee brace has undergone technical improvements; other orthoses described, but not regarding dental occlusion or any other side-effects	CA/CA^13^	2/low

CA – cannot apply: LoE impossible to establish due to poor reporting and/or missing data; JBI – Joanna Briggs Institute [49], [50]; ES – electrical stimulation; LESS – lateral electrical surface stimulation; NA – not addressed: such types of SRs are not listed in the OCEBM hierarchy; NNT – number needed to treat; OCEBM – Oxford Centre for Evidence Based Medicine [47], [48]; OMT – osteopathic manipulative treatment;

1 for details of the designs of included studies see Table 3; ^2^the detailed appraisal, with an elaboration, is in Table 1; ^3^Cochrane review; ^4^subsequent updates, as explained with Tables 1, 2, 3 and S1; ^5^one prospective controlled study included; therefore the OCEBM level was graded down one level, according to the classification rules; ^6^one pilot RCT on 6 IS patients included in the review; therefore the OCEBM level was graded down one level; ^7^one case study on AIS included in the review; therefore the OCEBM level was graded down one level;^ 8^for details regarding AMSTAR score of this SR see Table 1; ^9^scored 1b in the JBI classification, but the review was difficult to classify, as it included experimental, observational studies, and SRs; ^10^various study designs included, see Table 3; ^11^it was impossible to classify this review, as the authors did not report which specific study designs (except that the included studies were prospective or retrospective) were included or excluded; ^12^first (former) OCEBM classification; ^13^no study designs of included studies provided.

## Discussion

A brief summary of evidence from the reviews that were included is provided below.

### Scoliosis-specific exercises (SSE)

#### Romano et al. (2012) [33] high quality, AMSTAR score 9/11, 1/1a level of evidence SR

A recent (2012), rigorous Cochrane review [33] provided no convincing evidence from RCTs for or against these interventions in terms of curve progression as a primary outcome, and no evidence of risks or side effects from performing scoliosis-specific exercises.

#### Lower quality, lower level of evidence SRs [51]–[54], AMSTAR scores 4, 6, 2 and 1

A series of three other low to moderate quality SRs [51]–[53] recommended the use of SSE exercises based on level 1b evidence. Conversely another recent (2012), though very low quality SR [54] concluded that there was no evidence to support their use.

### Manual therapies

#### Posadzki et al. (2013) [7] higher quality SR, AMSTAR score 7/11

A recent (2013) SR [7] found one high quality RCT showing no evidence to support osteopathic manual therapy as an effective treatment for mild AIS.

#### Low quality reviews [6], [55], [56], AMSTAR scores 2, 3 and 4

Two other SRs [6], [55], though of lower quality, and lower level of evidence, provided similar conclusions. Conversely another low quality SR by McKennedy et al. [56] reported “very promising” findings from one pilot RCT.

### Bracing

#### Negrini et al. (2010) [16] high quality, AMSTAR score 9/11, 1/1a level of evidence SR

A Cochrane review from 2010 [16] found very low quality evidence supporting the effectiveness of bracing in reducing curve progression, and low quality evidence favoring hard braces as compared to soft braces. The update of this SR currently under review (JB-S, personal communication) found low to very low quality of evidence in favor of effectiveness of bracing in terms of reducing curve progression, with quality of life not highly impacted by bracing according to these studies.

#### Lenssick et al. (2005) [37] moderate quality SR, AMSTAR score 5/11

In a moderate quality SR of prospective controlled trials from 2005 [37], Lennsick et al. concluded, that due to the lowpower, weak methodological quality and clinical heterogeneity of the included studies, drawing firm conclusions was impossible. However the effectiveness of bracing and SSE treatments in reducing curve progression appeared promising. The authors did not formulate such claims with regards to electrical stimulation however. This SR scored 5 out of 11 with AMSTAR (moderate quality SR) although crucial elements of a SR were clearly reported within this review. Further although the assessment of publication bias was discussed in the paper the actual data was missing. Additionally even though a comprehensive search process was reported, this did not fully meet AMSTAR criteria [44]–[46].

#### Low quality reviews [17], [25], [26], [36], [43], [57], [58], AMSTAR scores 4, 2 and 1

The remaining SRs that met the inclusion criteria [17], [25], [26], [36], [43], [57], [58], were of low, or very low [17], [36] quality, and were classified using the OCEBM [47], [48] and JBI criteria [49], [50] as evidence of lower levels (Table 6).

The first SR on the conservative treatment of AIS by Focarile et al., that was found, dates back to 1991 [58]. It achieved an AMSTAR score of 4 and the conclusions of this review supported the use of braces. The meta-analysis by Rowe et al. from 1997 [26], evaluated different programs of bracing and of LESS. The results indicated that braces were effective only if they were worn 23 hours a day, but the results demonstrated no significant differences between LESS and observation. The Rowe et al. SR achieved only an AMSTAR score of 2. This was quite unexpected and remarkable, as this review, published in 1997, was based on evidence found within a textbook! What was even more surprising was the fact that the review was then used as a basis for producing the guidelines and recommendations [42], including the 2004 US Preventive Services Task Force recommendations, still current in 2014 [36]. A review published after this in 2008 [17] by Weiss and Goodall, evaluated the effectiveness of different methods of non-surgical treatment individually; first bracing, and then the outpatient and inpatient rehabilitation of AIS (these included SSEs typically used in Europe). This paper suggested that inpatient rehabilitation was effective but only achieved a score of 1 with AMSTAR.

The lowest quality SR by Sanders et al. [36] (2012) suggested that bracing may reduce the need for surgery, but other SRs [33], [43], [57] were not so convincing in their conclusions. Two of the SRs also considered patient-centered outcomes [43], [57] but found no firm evidence that bracing may negatively influence the quality of a patient’s life. The SR by Davies et al. [43] from 2011 further questioned the cost – effectiveness balance of bracing considering that there was no credible evidence of its effectiveness. Interestingly, their conclusions as to the quality of the available evidence were similar not only to those reported 20 years earlier in the 1991 review by Focarile et al. [58] but also to those from the high [33] and low [57] quality recent SRs by Negrini et al. and Maruyama et al.

### Usual physical activity

#### Green et al. (2009) [59] low quality review, AMSTAR score 4/11

One low quality review [59] (that scored 4 on AMSTAR), comprised five primary studies as well as six narrative reviews. A comparison of the study findings as well as different recommendations, found within this review provides cautious (grade D, Oxford CEBM) recommendations for the participation of AIS patients in sports who were either meaningfully observed or treated with braces. This paper however, rather than providing findings from a so called ‘systematic review’ primarily summarizes opinions formulated by undertaking a more biased narrative review and discusses findings from individual observational studies (three case-control studies, a survey and a case report), both of which provided low quality evidence of level 3 and 4 and 5, respectively [47]–[50]. Whilst this review provided information on the levels of evidence of the primary papers included, it did not sufficiently nor rigorously assess the methodological quality of these studies.

### Adverse effects

Two low quality reviews that addressed the adverse effects of brace wear were found by Li et al. [60] and Saccucci et al. [61], from 2008 and 2011, respectively. The 2008 review (AMSTAR score 3), based on the findings from five observational studies (one cross-sectional, one case-controlled, and three uncontrolled follow-up studies)concluded that there was no convincing evidence to support the assumption that brace wear may be associated with the loss of bone mineral density. The other review, by Saccucci et al. (AMSTAR score 2) included a narrative report on a case study from 1969. The authors suggested that there was an association between wearing the original Milwaukee brace (with a jaw support) and malocclusion. However, these claims now have only a historical meaning, as subsequently a large clinical controlled trial published in 1972 (also reported by Sacucci et al.), showed no such adverse effects associated with the use of the improved, thoraco-lumbo-sacral (TLSO) and soft (SpineCor) braces as none of these types of braces have a jaw support. The review by Saccucci et al. was very haphazardly conducted and very poorly reported with no clear data on the correlation of bracing and dental occlusion that could be determined.

### Additional non-surgical interventions not addressed in SRs

Other non-surgical interventions were reported in the literature, e.g. chiropractic and complementary and alternative medicine methods (acupuncture, Pilates exercises, or herbal therapy) [6]–[8], however no secondary analyses addressing those approaches were found.

### Quality analyses

As reported in detail in the Results section, and in Tables 3–5, the methodological quality of the majority (16 out of 21) of the retrieved SRs was disappointingly low, regardless of the limitations that were independent from the study authors – such as the number, quality, design and comparability of eligible primary studies. In many SRs there was no second independent reviewer and blind study selection and/or data extraction, no lists of included and excluded studies, no comprehensive search for evidence, and, perhaps most importantly, no quality assessment of included studies conducted. The reviews instead reported only (more or less detailed) study characteristics. In some of the reviews the level of evidence hierarchy classification (categories of studies) were reported as a quality assessment suggesting perhaps a lack of knowledge amongst clinicians conducting SRs regarding systematic review methodology. Further, a number of excluded reviews (Table S2) were called “systematic” but actually comprised only a structured and systematic literature search, and then presented a narrative discussion of a few papers of diverse designs. The only SR with a meta-analysis by Rowe et al. [26] was seriously flawed methodologically (AMSTAR score 2 out of 11, Table 3) with findings and conclusions that were biased (Table 5). This review (as well as the SR by Focarile et al. [58]) did not differentiate between juvenile and adolescent IS. As these conditions differ in their clinical characteristics therefore their findings can be regarded as even less credible.

The low methodological quality found within a large proportion of the so called systematic reviews in this area, is in general very disappointing, especially when comparing these findings to recent overviews that have confirmed the good methodological quality of systematic reviews within the areas of rehabilitation [64] and orthopedics [65]. These results suggest that not only are good RCTs and prospective studies with a control group needed, but also as important, there is a fundamental need to improve the quality not only of conducting, but also writing and presenting systematic reviews in the subject matter addressed within this paper. It would also be suggested that education in the conduct and presentation of state of the art systematic reviews are prioritized within medical and health care education.

#### Quality of reviews vs quality of reporting

The objective of this current paper was to evaluate the methodological **quality of systematic reviews**, not the **quality of reporting.** However, it must also be acknowledged that clear reporting does not necessarily result in a high quality review. Some reviews were clearly reported, but nonetheless had a number of methodological limitations.

The high quality reviews [16], [33] did not meet the AMSTAR criteria [44]–[46], regarding the assessment of the likelihood of publication bias as well as the criteria on the reporting of conflicts of interest statements within individual primary studies. These issues indicate minor limitations in reporting, rather than the processes undertaken to conduct and develop the systematic review, in terms of the AMSTAR criteria [45]. The moderate quality reviews [7], [37], [52] generally met the substantial criteria for a valid systematic review, but did not meet some of the criteria for comprehensively conducting and reporting (Tables 3–5), such as providing the ‘a priori design’ of the review (e.g. in a SR protocol), comprehensive searching, regardless of the publication status (gray literature) and language restrictions, as well as providing lists of included and excluded publications. The lower quality SRs were either clearly reported, but appeared less careful with the reporting of the methodological process undertaken [25], [43], [52], were haphazardly undertaken [61], had language limitations [58], [61] and/or were written in a way that did not follow contemporary reporting criteria [26], [58].

### Types of reviews and outcome measures

Although systematic reviews of uncontrolled observational studies, especially of retrospective designs may be developed according to standard criteria [66], this does not eliminate the bias resulting from the methodological constraints of the included studies. Another issue is the type and meaning of primary and secondary outcome measures. Curve progression as a criterion of treatment success is considered a primary outcome measure within many SRs (e.g. [6], [25], [26], [51]–[53], Table 4). In point of fact however, primary, patient-centered outcomes, (considered in the available Cochrane reviews [16], [33] as well as in a number of other SRs [43], [57]) are outcomes that are of most concern to the patients themselves; these include such outcomes as for example neuromotor control, balance, back pain, or respiratory function. Curve progression, in terms of patient-centered outcomes, is regarded by the Cochrane Back Research Group (CBRG) as a surrogate, or as a secondary end-point or outcome measure. The effects of brace treatment have to date been controversial as to the impact on patients’ and families’ quality of life and other adverse events [12], [67]. Furthermore, a cost-utility analyses indicated that outcome measures need to be patient-centered and that both outcomes and costs are measured and assessed in the long-term [68], [69].

### Quality of reviews and levels of evidence

An issue not covered through the appraisal with the AMSTAR tool – the research design of primary studies included within a review – necessarily influences the level of evidence derived from a SR, and is addressed and interpreted differently within different classifications of the hierarchy of levels of evidence currently available. Significant difficulties were encountered when trying to categorize the levels of evidence (LoE) of the SRs that were included. This was due to the very unclear characteristics of the large majority of the SRs in terms of the study designs that were included for analysis (Table 5).

The current Oxford CEBM classification [47], [48] categorizes SRs of RCTs as a step 1 (or level 1) evidence for questions regarding treatment benefits and common harms. However it does not list SRs of other types of research designs besides RCTs for treatment benefits, and only lists SRs of nested case-control studies as step 2 (level 2) of evidence. Conversely the latest Joanna Briggs Institute’s “New Levels of Evidence” document [49], [50] classifies SRs of different types of studies with the highest sub-level for each of 5 levels of evidence, where a SR comprised of RCTs is allocated a level 1a, and SRs of expert opinion (!) is considered to be a level 5a of evidence (although it is unclear to the authors of this paper how a SR of expert opinion should be conducted).

Furthermore it is worth noting the fact that, a systematic review that includes either an inferential statistical analysis (meta-analysis), or alternatively is a qualitative systematic review, is not a criterion that influences the current levels of evidence achieved in either the OCEBM or the JBI classifications. In fact, the three quantitative reviews by Dolan and Weinstein [25], Focarile et al. [58] and Rowe et al. [26], which included pooled data syntheses (meta-analysis) and were included in the present study, all scored as low quality SRs with AMSTAR while the most rigorous, high level evidence reviews of clinical trials [7], [16], [33] did not include any meta-analyses. As a point in fact very few SRs (those of moderate and high methodological quality (Table 3) – considered the research study designs of included studies as important criteria for the conduction of a valid and reliable SR [70].

### Comparisons with other studies

No overview of systematic reviews addressing the effect of non-surgical interventions on patients with AIS could be found. However, an analysis of one of the included SRs [6] was reported in an overview of systematic reviews addressing manual therapy in various pediatric conditions [63]. Additionally brief critiques of one of the included SRs [26] were found in two of the SRs that were analyzed [22], [37]. Finally, critical abstracts of two of the included SRs [26], [51] are provided in the DARE database. Generally, the assumptions and analyses within the DARE database correspond with the findings of this study.

### Limitations of the study

As is typical for systematic overviews of systematic reviews, an analysis of the overall methodological quality of all the included systematic reviews (not the primary studies included in the reviews) was conducted within this study. Thus, information regarding the design and methodology of individual primary studies were, except in very unclear cases, based on the quality appraisals reported within the systematic reviews that were included and analyzed. With the exception of one review [43], the authors were not contacted.

#### Evidence from very recent primary studies and unpublished updated SR

Recently, the first multicenter randomized controlled BrAIST trial evaluating the effectiveness of bracing on AIS [23], as well as a randomized controlled trial on the effectiveness of a scoliosis-specific exercise program [71], both found the interventions to be effective. Conversely a very recent prospective controlled trial by Sanders et al. (2014) [72] claimed that only highly compliant patients may avoid surgery through brace wear. Furthermore, an update of a Cochrane review considered in this paper [33], currently under review (JB-S, personal communication), demonstrates improvements in terms of the evidence-base in this subject matter, however the Negrini (2014, unpublished) Cochrane brace review included seven prospective trials (five RCTs) of different quality, which reached different conclusions. These add to, and seem to alter, the evidence-base regarding brace and exercise treatments. However, the assessment of methodological quality of primary and unpublished studies was beyond the scope of this study.

### Conclusions

- The methodological quality of systematic reviews in the area of non-surgical interventions for of AIS is generally low;
- Findings from higher quality reviews that consider numerous outcome measures, indicate that generally there is insufficient evidence to enable researchers and clinicians as well as service users to make a judgment on whether non-surgical interventions in AIS are effective;
- Individual, highly cited and older reviews, demonstrating the effectiveness of rigorously applied braces and physiotherapy, were found to be of low methodological quality; so it is unclear to what extent the results of these reviews are valid;
- Readers need to be aware that papers entitled as systematic reviews may not necessarily meet the criteria to be classified as systematic reviews or in other words, papers entitled as systematic reviews need to be considered in terms of their methodological rigor; otherwise they may be low quality sources of evidence that are mistakenly regarded as high quality ones.

To the authors’ best knowledge, this is the first comprehensive, explicit and systematic overview of systematic reviews addressing diverse non-surgical interventions for adolescents with idiopathic scoliosis. The authors believe that the findings of this overview will be of significant benefit to patients and parents, clinicians, researchers and commissioners of health services in this field.

## Supporting Information

Table S1 **List of included reviews.**
(DOCX)

Table S2 **List of excluded papers.**
(DOCX)

Table S3 **Details of the electronic search and selection process.**
(DOCX)

Protocol S1 **PROSPERO protocol.**
(PDF)

Checklist S1 **PRISMA 2009 Checklist.**
(DOCX)

## References

[1] Bettany-Saltikov J, Parent E, Romano M, Villagrasa M, Negrini S (2014) Physiotherapeutic scoliosis-specific exercises for adolescents with idiopathic scoliosis. Eur J Phys Rehabil Med 50: 111–121. 24525556

[2] Hawes MC (2003) The use of exercises in the treatment of scoliosis: an evidence-based critical review of the literature. Pediatr Rehabil 6: 171–182. 14713583 10.1080/0963828032000159202

[3] Negrini S, Aulisa AG, Aulisa L, Circo AB, de Mauroy JC, et al (2012) 2011 SOSORT guidelines: Orthopaedic and rehabilitation treatment of idiopathic scoliosis during growth. Scoliosis 7: 3 Available: http://www.scoliosisjournal.com/content/7/1/3 Accessed 2014 Feb 10.. 22264320 10.1186/1748-7161-7-3 PMC3292965

[4] Zaina F, De Mauroy JC, Grivas T, Hresko MT, Kotwicki T, et al (2014) Bracing for scoliosis in 2014: state of the art. Eur J Phys Rehabil Med 50: 93–110. 24622051

[5] Horne JP, Flannery R, Usman S (2014) Adolescent idiopathic scoliosis: diagnostics and management. Am Fam Physician 89: 193–198. 24506121

[6] Romano M, Negrini S (2008) Manual therapy as a conservative treatment for adolescent idiopathic scoliosis. Scoliosis 3: 2 Available: http://www.scoliosisjournal.com/content/3/1/2 Accessed 2012 Nov 28.. 18211702 10.1186/1748-7161-3-2 PMC2262872

[7] Posadzki P, Lee MS, Ernst E (2013) Osteopathic manipulative treatment for pediatric conditions: a systematic review. Pediatrics 132: 140–152. 23776117 10.1542/peds.2012-3959

[8] Zarzycka M, Rozek K, Zarzycki M (2009) Alternative methods of conservative treatment of idiopathic scoliosis. Ortop Traumatol Rehabil 11: 396–412. 19920282

[9] Dickson RA (1999) Spinal deformity – adolescent idiopathic scoliosis. Nonoperative treatment. Spine 24: 2601–2606. 10635523 10.1097/00007632-199912150-00007

[10] Reamy BV, Slakery JB (2001) Adolescent idiopathic scoliosis: review and current concepts. Am Fam Physician 64: 111–116. 11456428

[11] Lonstein JE (1994) Adolescent idiopathic scoliosis. Lancet 344: 1407–1412. 7968079 10.1016/s0140-6736(94)90572-x

[12] Weinstein SL, Dolan LA, Cheng JCY, Danielson A, Morcuende JA (2008) Adolescent idiopathic scoliosis. Lancet 371: 1527–1537. 18456103 10.1016/S0140-6736(08)60658-3

[13] Skaggs DL, Bassett GS (1996) Adolescent idiopathic scoliosis: an update. Am Fam Physician 53: 2327–2335. 8638509

[14] Weiss HR, Negrini S, Hawes MC, Rigo M, Kotwicki T, et al (2006) Physical exercises in the treatment of idiopathic scoliosis at risk of brace treatment – SOSORT consensus paper 2005. Scoliosis 1: 6 Available: http://www.scoliosisjournal.com/content/1/1/6 Accessed 2014 Feb 11.. 16759360 10.1186/1748-7161-1-6 PMC1481573

[15] Weiss HR (2003) Rehabilitation of adolescent patients with scoliosis – what do we know? A review of the literature. Pediatr Rehab 6: 183–194. 10.1080/13638490310001636790 14713584

[16] Negrini S, Minozzi S, Bettany-Saltikov J, Zaina F, Chockalingam N, et al.. (2010) Braces for idiopathic scoliosis in adolescents. Cochrane Database Syst Rev CD006850. 10.1002/14651858.CD006850.pub2 20091609

[17] Weiss HR, Goodall D (2008) The treatment of adolescent idiopathic scoliosis (AIS) according to present evidence. A systematic review. Eur J Phys Rehabil Med 44: 177–193. 18418338

[18] Cassella M, Hall JE (1991) Current treatment approaches in the nonoperative and operative management of adolescent idiopathic scoliosis. Phys Ther 71: 897–909. 1946624 10.1093/ptj/71.12.897

[19] Altaf F, Gibson A, Dannawi Z, Noordeen H (2013) Adolescent idiopathic scoliosis. BMJ 346: f2508. 23633006 10.1136/bmj.f2508

[20] Hresko MT (2013) Idiopathic scoliosis in adolescents. N Engl J Med 368: 834–841. 23445094 10.1056/NEJMcp1209063

[21] Schiller JR, Thakur NA, Eberson CP (2010) Brace management in adolescent idiopathic scoliosis. Clin Orthop Relat Res 468: 670–678. 19484317 10.1007/s11999-009-0884-9 PMC2816747

[22] Stokes OM, Luk KD (2013) The current status of bracing for patients with adolescent idiopathic scoliosis. Bone Joint J 95-B: 1308–1316. 24078524 10.1302/0301-620X.95B10.31474

[23] Weinstein SL, Dolan LA, Wright JG, Dobbs MB (2013) Effects of bracing in adolescents with idiopathic scoliosis. N Engl J Med 369: 1512–1521. 24047455 10.1056/NEJMoa1307337 PMC3913566

[24] Scoliosis Research Society (2014) Adolescent Idiopathic Scoliosis. Treatment. Available: http://www.srs.org/professionals/conditions_and_treatment/adolescent_idiopathic_scoliosis/treatment.htm. Accessed 2014 May 1.

[25] Dolan LA, Weinstein SL (2007) Surgical rates after observation and bracing for adolescent idiopathic scoliosis: an evidence-based review. Spine 32: S91–S100. 17728687 10.1097/BRS.0b013e318134ead9

[26] Rowe DE, Bernstein SM, Riddick MF, Adler F, Emans JB, Gardner-Bonneau D, et al (1997) A meta-analysis of the efficacy of non-operative treatments for idiopathic scoliosis. J Bone Joint Surg Am 79: 664–674. 9160938 10.2106/00004623-199705000-00005

[27] Mo F, Cunningham ME (2011) Pediatric scoliosis. Curr Rev Musculoskelet Med 4: 175–182. 22021016 10.1007/s12178-011-9100-0 PMC3261245

[28] Disability and Rehabilitation journal (2008) Thematic issue: Rehabilitation of adolescent idiopathic scoliosis. Disabil Rehabil 30: 731–817.18432431

[29] Hasson S (2011) Special issue: Scoliosis and Evidence-based Practice. Physiother Theor Pract 271. 10.3109/09593985.2011.537613 21198401

[30] National Institute for Health Research Health Technology Assessment Programme (2012) Active treatment for idiopathic adolescent scoliosis (ACTIvATeS): a pilot randomised controlled trial. Protocol number ISRCTN90480705. Available: http://www.nets.nihr.ac.uk/__data/assets/pdf_file/0017/55403/PRO-10-38-03.pdf. Accessed 2014 Feb 20.

[31] University of Alberta (2012) Schroth Exercise Trial for Scoliosis (SETS). ClinicalTrials.gov [Internet]. Bethesda (MD): National Library of Medicine (US). NLM Identifier NCT01610908. Available: http://clinicaltrials.gov/show/NCT01610908. Accessed 2014 Feb 20.

[32] Karolinska Institutet (2012) Trial on Three Treatments for Scoliosis (CONTRAIS). ClinicalTrials.gov [Internet]. Bethesda (MD): National Library of Medicine (US). NLM Identifier NCT01761305. Available: http://clinicaltrials.gov/show/NCT01761305. Accessed 2014 Feb 20.

[33] Romano M, Minozzi S, Bettany-Saltikov J, Zaina F, Chockalingam N, et al (2012) Exercises for adolescent idiopathic scoliosis. Cochrane Database Syst Rev 8: CD007837. 10.1002/14651858.CD007837.pub2 PMC7386883 22895967

[34] Sponseller PD (2011) Bracing for adolescent idiopathic scoliosis in practice today. J Pediatr Orthop 31(1 Suppl): S53–S60. 10.1097/BPO.0b013e3181f73e87 21173620

[35] US Preventive Services Task Force (2004) Screening for idiopathic scoliosis in adolescents: a brief evidence update. AHRQ Pub. No.05-0568-B. Available: http://www.uspreventiveservicestaskforce.org/3rduspstf/scoliosis/scolioup.pdf. Accessed 2014 July 1.

[36] Sanders JO, Newton PO, Browne RH, Herring AJ (2012) Bracing in adolescent idiopathic scoliosis, surrogate outcomes, and the number needed to treat. J Pediatr Orthop 32 Suppl 2: S153–S157. 22890455 10.1097/BPO.0b013e31825199e5

[37] Lenssinck ML, Frijlink AC, Berger MY, Bierman-Zeinstra SM, Verkerk K, et al (2005) Effect of bracing and other conservative interventions in the treatment of idiopathic scoliosis in adolescents: a systematic review of clinical trials. Phys Ther 12: 1329–1339. 16305271

[38] Moher D, Liberati A, Tetzlaff J, Altman DG (2009) The PRISMA Group (2009) *P*referred *R*eporting *I*tems for *S*ystematic Reviews and *M*eta-*A*nalyses: The PRISMA Statement. PLoS Med 6(6): e1000097. 19621072 10.1371/journal.pmed.1000097 PMC2707599

[39] Liberati A, Altman DG, Tetzlaff J, Mulrow C, Gøtzsche PC, et al (2009) The PRISMA statement for reporting systematic reviews and meta-analyses of studies that evaluate healthcare interventions: explanation and elaboration. BMJ 2009 339: b2700. 10.1136/bmj.b2700 PMC2714672 19622552

[40] Hartling L, Chisholm A, Thomson D, Dryden DM (2012) A descriptive analysis of overviews of reviews published between 2000 and 2011. PLoS ONE 7(11): e49667. 23166744 10.1371/journal.pone.0049667 PMC3499476

[41] The Cochrane Collaboration (2005) Glossary of Terms in The Cochrane Collaboration. Version 4.2.5. Available: http://www.cochrane.org/sites/default/files/uploads/glossary.pdf. Accessed 2010 May 20.

[42] Płaszewski M, Bettany-Saltikov J (2014) Are current scoliosis school screening recommendations evidence-based and up to date? A best evidence synthesis umbrella review. Eur Spine J [Epub ahead of print]: DOI 10.1007/s00586-014-3307-x. 10.1007/s00586-014-3307-x 24777669

[43] Davies E, Norvell D, Hermsmeyer J (2011) Efficacy of bracing versus observation in the treatment of idiopathic scoliosis Evid Based Spine Care J. 2: 25–34. 10.1055/s-0030-1267102 PMC3621850 23637679

[44] Shea BJ, Grimshaw JM, Wells GA, Boers M, Andersson N, et al (2007) Development of AMSTAR: a measurement tool to assess the methodological quality of systematic reviews. BMC Med Res Methodol 7: 10. 17302989 10.1186/1471-2288-7-10 PMC1810543

[45] Shea BJ, Bouter LM, Peterson J, Boers M, Andersson N, et al (2007) External validation of a measurement tool to assess systematic reviews (AMSTAR). PLoS ONE 2(12): e1350. 18159233 10.1371/journal.pone.0001350 PMC2131785

[46] Popovic I, Windsor B, Jordan V, Showell M, Shea B, et al (2012) Methodological quality of systematic reviews in subfertility: a comparison of two different approaches. PLoS One 7(12): e50403. 23300526 10.1371/journal.pone.0050403 PMC3532502

[47] OCEBM Levels of Evidence Working Group (2011) The Oxford 2011 Levels of Evidence. Oxford Centre for Evidence-Based Medicine. Available: http://www.cebm.net/index.aspx?o=5653. Accessed 2014 Mar 15.

[48] Howick J, Chalmers I, Glasziou P, Greenhalgh T, Heneghan C, et al. (2011) Explanation of the 2011 Oxford Centre for Evidence-Based Medicine (OCEBM) Levels of Evidence (Background Document). Oxford Centre for Evidence-Based Medicine. Available: http://www.cebm.net/index.aspx?o=5653. Accessed 2014 Mar 15.

[49] The Joanna Briggs Institute Levels of Evidence and Grades of Recommendation Working Party (2014) New JBI Levels of Evidence. The Joanna Briggs Institute. Available: http://joannabriggs.org/assets/docs/approach/JBI-Levels-of-evidence_2014.pdf. Accessed 2014 Mar 15.

[50] The Joanna Briggs Institute Levels of Evidence and Grades of Recommendation Working Party (2014) Supporting Document for the Joanna Briggs Institute Levels of Evidence and Grades of Recommendation. The Joanna Briggs Institute. Available: http://joannabriggs.org/assets/docs/approach/Levels-of-Evidence-SupportingDocuments.pdf. Accessed 2014 Mar 15.

[51] Negrini S, Antonini G, Carabalona R, Minozzi S (2003) Physical exercises as a treatment for adolescent idiopathic scoliosis. A systematic review. Pediatric Rehabil 3–4: 227–235. 10.1080/13638490310001636781 14713590

[52] Negrini S, Fusco C, Minozzi S, Atanasio S, Zaina F, et al (2008) Exercises reduce the progression rate of adolescent idiopathic scoliosis: results of a comprehensive systematic review of the literature. Disabil Rehab 30: 772–785. 10.1080/09638280801889568 18432435

[53] Fusco C, Zaina F, Atanasio S, Romano M, Negrini A, et al (2011) Physical exercises in the treatment of adolescent idiopathic scoliosis: an updated systematic review. Physiother Theory Pract 1: 80–114. 10.3109/09593985.2010.533342 21198407

[54] Mordecai SC, Dabke HV (2012) Efficacy of exercise therapy for the treatment of adolescent idiopathic scoliosis: a review of the literature. Eur Spine J 21: 382–389. 22065168 10.1007/s00586-011-2063-4 PMC3296853

[55] Gleberzon BJ, Arts J, Mei A, McManus EL (2012) The use of spinal manipulative therapy for pediatric health conditions: a systematic review of the literature. J Can Chiropr Assoc 56: 128–141. 22675226 PMC3364062

[56] McKennedy K, Sinclair Elder A, Elder C, Hutchins A (2013) Myofascial release as a treatment for orthopaedic conditions: a systematic review. J Athl Training 48: 522–527. 10.4085/1062-6050-48.3.17 PMC3718355 23725488

[57] Maruyama T, Grivas TB, Kaspiris A (2011) Effectiveness and outcomes of brace treatment: A systematic review. Physiother Theor Pract 27: 26–42. 10.3109/09593985.2010.503989 21198404

[58] Focarile FA, Bonaldi A, Giarolo MA, Ferrari U, Zilioli E, et al (1991) Effectiveness of non-surgical treatment for idiopathic scoliosis. Overview of available evidence. Spine 16: 395–401. 1828625 10.1097/00007632-199104000-00001

[59] Green BN, Johnson CD, Moreau W (2009) Is physical activity contraindicated for individuals with scoliosis? A systematic literature review. J Chiropr Med 8: 25–37. 19646383 10.1016/j.jcm.2008.11.001 PMC2697577

[60] Li XF, Li H, Liu ZD, Dai LY (2008) Low bone mineral status in adolescent idiopathic scoliosis. Eur Spine J 17: 1431–1440. 18751741 10.1007/s00586-008-0757-z PMC2583185

[61] Saccucci M, Tettamanti L, Mummolo S, Polimeni A, Festa F, et al (2011) Scoliosis and dental occlusion: a review of the literature. Scoliosis 6: 15 Available: http://www.scoliosisjournal.com/content/6/1/15 Accessed 2012 Dec 2.. 21801357 10.1186/1748-7161-6-15 PMC3162939

[62] Weiss HR (2012) Intervention studies on scoliosis – review of the reviews. Polish Annals of Medicine 19: 72–83.

[63] Posadzki P, Ernst E (2011) Spinal manipulation: an update of a systematic review of systematic reviews. NZMJ 124: 55–71. 21952385

[64] Gianola S, Gasparini M, Agostini M, Castellini G, Corbetta D, et al (2013) Survey of the reporting characteristics of systematic reviews in rehabilitation. Phys Ther 93: 1456–1466. 23744458 10.2522/ptj.20120382

[65] Gagnier JJ, Kellam PJ (2013) Reporting and methodological quality of systematic reviews in the orthopaedic literature. J Bone Joint Surg Am 95: e771–e777. 23780547 10.2106/JBJS.L.00597

[66] Stroup DF, Berlin JA, Morton SC, Olkin I, Williamson GD, et al (2000) Meta-analysis of observational studies in epidemiology: a proposal for reporting. JAMA 283: 2008–2012. 10789670 10.1001/jama.283.15.2008

[67] Falk B, Rigby A, Akseer N (2014) Adolescent idiopathic scoliosis: the possible harm of bracing and the likely benefit of exercise. Spine J [Epub ahead of print]: DOI 10.1016/j.spinee.2014.05.00.

[68] Rihn JA, Berven S, Allen T, Phillips FM, Currier BL, et al (2009) Defining value in spine care. Am J Med Qual 24: 4S–14S. 19890180 10.1177/1062860609349214

[69] Kepler CK, Wilkinson SM, Radcliff KE, Vaccaro AR, Anderson DG (2012) Cost-utility analysis in spine care: a systematic review. Spine J 12: 676–690. 22784806 10.1016/j.spinee.2012.05.011

[70] Centre for Reviews and Dissemination (2008) Systematic Reviews. CRD’s guidance for undertaking systematic reviews in health care. University York. 9, 34. Available: http://www.york.ac.uk/inst/crd/index_guidance.htm. Accessed 2011 Oct 23.

[71] Monticone M, Ambrosini E, Cazzaniga D, Rocca B, Ferrante S (2014) Active self-correction and task-oriented exercises reduce spinal deformity and improve quality of life in subjects with mild adolescent idiopathic scoliosis. Results of a randomised controlled trial. Eur Spine J [Epub ahead of print]: DOI 10.1007/s00586-014-3241-y. 10.1007/s00586-014-3241-y 24682356

[72] Sanders JO, Newton PO, Browne RH, Katz DE, Birch JG, et al (2014) Bracing for idiopathic scoliosis: how many patients require treatment to prevent one surgery? J Bone Joint Surg Am 96: 649–653. 24740661 10.2106/JBJS.M.00290

