# Supplementary material for: Non-Surgical Interventions for Adolescents with Idiopathic Scoliosis: An Overview of Systematic Reviews
Source: PLoS One. 2014 Oct 29;9(10):e110254. doi: 10.1371/journal.pone.0110254 (PMC4213139; doi:10.1371/journal.pone.0110254)
Supplement: Table S1 — List of included reviews. (DOCX) [file pone.0110254.s001.docx]

Table S1. List of included reviews. Reasons for inclusion in the overview are explained if not clear.

| **paper** | **reason(s) for inclusion** |
| --- | --- |
| Focarile FA, Bonaldi A, Giarolo MA, Ferrari U, Zilioli E, Ottaviani C. Effectiveness of nonsurgical treatment for idiopathic scoliosis. Overview of available evidence. Spine 1991; 16: 395-401. |  |
| Rowe DE, Bernstein SM, Riddick MF, Adler F, Emans JB, Gardner-Bonneau D. A meta-analysis of the efficacy of non-operative treatments for idiopathic scoliosis. J Bone Joint Surg Am 1997; 79: 664-74. |  |
| Lenssinck ML, Frijlink AC, Berger MY, Bierman-Zeinstra SM, Verkerk K, Verhagen AP. Effect of bracing and other conservative interventions in the treatment of idiopathic scoliosis in adolescents: a systematic review of clinical trials. Phys Ther 2005;12:1329-39 |  |
| Dolan LA, Weinstein SL. Surgical rates after observation and bracing for adolescent idiopathic scoliosis: an evidence-based review. Spine 2007;32:S91-S100. | surgery – an outcome, not an intervention analysed |
| Romano M, Negrini S. Manual therapy as a conservative treatment for adolescent idiopathic scoliosis. Scoliosis 2008;3:2 |  |
| Weiss HR, Goodall D. The treatment of adolescent idiopathic scoliosis (AIS) according to present evidence. A systematic review. Eur J Phys Rehabil Med 2008;44:177-93 |  |
| Li XF, Li H, Liu ZD, Dai LY. [Low bone mineral status in adolescent idiopathic scoliosis.](http://www.ncbi.nlm.nih.gov/pubmed/18751741) Eur Spine J 2008;17:1431-40 | analysis of effects of bracing on bone density |
| Green BN, Johnson CD, Moreau W. Is physical activity contraindicated for individuals with scoliosis? A systematic literature review. J Chiropr Med 2009;8:25-37 | this SR regards usual or sports physical activities, not scoliosis-specific exercise therapy programmes; however, the paper regards interventions of interest for physiotherapists |
| Negrini S, Minozzi S, Bettany-Saltikov J, Zaina F, Chockalingam N, Grivas TB, Kotwicki T, Maruyama T, Romano M, Vasiliadis ES. Braces for idiopathic scoliosis in adolescents. Cochrane Database Syst Rev 2010;CD006850 |  |

Table S1. Cont.

| **paper** | **reason(s) for inclusion** |
| --- | --- |
| Negrini S, Antonini G, Carabalona R, Minozzi S. Physical exercises as a treatment for adolescent idiopathic scoliosis. A systematic review. Pediatric Rehabil 2003;3-4:227-35.  (SR 1 of 3) | Consecutive series of systematic reviews:  the 2008 review is an update of the 2003 review, with a separate analysis of newly added studies, and with separate conclusions; the 2011 update cannot be analysed separately from the 2008 review: the authors cumulated studies included in both reviews (added 1 study in the 2011 review), refer to the methodology described in the 2008 review, and the characteristics of included studies are described in the 2008 paper; AMSTAR appraisals are done individually for all papers; study characteristics are provided in concert or separately, depending on how the authors addressed individual issues |
| Negrini S, Fusco C, Minozzi S, Atanasio S, Zaina F, Romano M. Exercises reduce the progression rate of adolescent idiopathic scoliosis: results of a comprehensive systematic review of the literature. Disabil Rehab 2008;30:772-85  (SR 2 of 3) |  |
| Fusco C, Zaina F, Atanasio S, Romano M, Negrini A, Negrini S. Physical exercises in the treatment of adolescent idiopathic scoliosis: an updated systematic review. Physiother Theory Pract 2011;1:80-114  (SR 3 of 3) |  |
| Saccucci M, Tettamanti L, Mummolo S, Polimeni A, Festa F, Salini V, Tecco S. Scoliosis and dental occlusion: a review of the literature. Scoliosis 2011;6:15 | systematic review regarding adverse effects of non-operative treatment: associations between Milwaukee brace wear and dental malformations |
| Davies E, Norvell D, Hermsmeyer J. Efficacy of bracing versus observation in the treatment of idiopathic scoliosis Evid Based Spine Care J 2011; 2:25–34. |  |

Table S1. Cont.

| **paper** | **reason(s) for inclusion** |
| --- | --- |
| Maruyama T, Grivas TB, Kaspiris A. Effectiveness and outcomes of brace treatment: A systematic review. Physiother Theor Pract 2011;27:26-42 |  |
| Mordecai SC, Dabke HV. Efficacy of exercise therapy for the treatment of adolescent idiopathic scoliosis: a review of the literature. Eur Spine J 2012;21: 382-9. |  |
| Gleberzon BJ, Arts J, Mei A, McManus EL. The use of spinal manipulative therapy for pediatric health conditions: a systematic review of the literature. J Can Chiropr Assoc 2012;56:128-41 |  |
| Romano M, Minozzi S, Bettany-Saltikov J, Zaina F, Chockalingam N, Kotwicki T, Maier-Hennes A, Negrini S. Exercises for adolescent idiopathic scoliosis. Cochrane Database of Systematic Reviews 2012;8:CD007837 |  |
| Sanders JO, Newton PO, Browne RH, Herring AJ. Bracing in adolescent idiopathic scoliosis, surrogate outcomes, and the number needed to treat. J Pediatr Orthop 2012;32 Suppl 2:S153-7 |  |
| McKennedy K, Sinclair Elder A, Elder C, Hutchins A. Myofascial release as a treatment for orthopaedic conditions: a systematic review. J Athl Training 2013;48:522-7 | the eligibility criterion was adult population, but a study on idiopathic scoliosis was included for analysis |
| Posadzki P, Lee MS, Ernst E. Osteopathic manipulative treatment for pediatric conditions: a systematic review. Pediatrics 2013;132:140-152 | an SR of RCTs; studies on idiopathic scoliosis included |

USPSTF – US Preventive Services Task Force; UK NSC – UK National Screening Committee; DARE – Database of Abstracts of Reviews of Interventions; SR – systematic review; RCT – randomised controlled trial
