# Supplementary material for: Non-Surgical Interventions for Adolescents with Idiopathic Scoliosis: An Overview of Systematic Reviews
Source: PLoS One. 2014 Oct 29;9(10):e110254. doi: 10.1371/journal.pone.0110254 (PMC4213139; doi:10.1371/journal.pone.0110254)
Supplement: Table S2 — List of excluded papers. (DOCX) [file pone.0110254.s002.docx]

Table S2. List of papers excluded after full text analysis, with rationale.

| **systematic reviews** | **reason(s) for exclusion** |
| --- | --- |
| Oakley PA, Harrison DD, Harrison DE, Haas JW. Evidence-based protocol for structural rehabilitation of the spine and posture: review of clinical biomechanics of posture (CBP®) publications. JCCA 2005;49:270-96 | scoliosis conservative treatment not an outcome of interest |
| Wind H, Gouttebarge V, Kuijer PPFM, Frings-Dresen MHW. Assessment of functional capacity of the musculoskeletal system in the context of work, daily living, and sport: a systematic review. J Occup Rehabil 2005;15:253-72 | a single study regarding scoliosis assessed for methodological quality, but not presented as did not meet the minimal inclusion quality criteria |
| Simpson R, Gemmell H. Accuracy of spinal orthopaedic tests: a systematic review. Chiropractic & Osteopathy 2006, 14:26 | not regarding intervention studies |
| Christensen ST, Hartvigsen J. Spinal curves and health: a systematic critical review of the epidemiological literature dealing with the associations between sagittal spinal curves and health. J Manipulative Physiol Ther 2008;31:690-714. | not specifically on scoliosis |
| Weiss HR. Adolescent idiopathic scoliosis (AIS) – an indication for surgery? A systematic review of the literature. Disabil Rehabil 2008;30:799-807 | conservative treatment not an inclusion criterion |
| Venning A, Eliott J, Wilson A, Kettler L. Understanding young peoples' experience of chronic illness: a systematic review. Int J Evid Based Healthc 2008;6:321-36 | systematic review of qualitative studies, with meta-synthesis; scoliosis, however, not regarded separately |
| Kepler CK, Wilkinson SM, Radcliff KE, Vaccaro AR, Anderson DG, Hilibrand AS, Albert TJ, Rihn JA. Cost-utility analysis in spine care: a systematic review. Spine J 2012;12:676-90. | one cost-utility analysis addressing adolescent idiopathic scoliosis included, but not regarding non-surgical interventions* |
| Kuo Y-L, Lee L-L. Prevalence and risk factors associated with spinal pain in adolescent computer users: a systematic review. JBI Library of Systematic Reviews, 2012;10:45. <http://joannabriggslibrary.org/index.php/jbisrir/article/view/26>. Accessed 16 March 2014 | scoliosis not an inclusion criterion |
| Rushton PR, Grevitt MP. Comparison of untreated adolescent idiopathic scoliosis with normal controls: a review and statistical analysis of the literature. Spine 2013;38:778-85 | not on treatment |

Table S2. Cont.

| **systematic reviews** | **reason(s) for exclusion** |
| --- | --- |
| Pinquart M, Shen Y. [Anxiety in children and adolescents with chronic physical illnesses: a meta-analysis.](http://www.ncbi.nlm.nih.gov/pubmed/21332786) Acta Paediatr 2011;100:1069-76 | not on treatment (moderating effect of chronic illness on different characteristics tested, but an effect of treatment not tested); scoliosis an inclusion criterion only in some of the reviews |
| Pinquart M, Shen Y. [Depressive symptoms in children and adolescents with chronic physical illness: an updated meta-analysis.](http://www.ncbi.nlm.nih.gov/pubmed/21088072) J Pediatr Psychol 2011;36:375-84 |  |
| Pinquart M, Teubert D. [Academic, physical, and social functioning of children and adolescents with chronic physical illness: a meta-analysis.](http://www.ncbi.nlm.nih.gov/pubmed/22173882) J Pediatr Psychol 2012;37:376-89 |  |
| Pinquart M. Body image of children and adolescents with chronic illness: A meta-analytic comparison with healthy peers. Body Image 2013;10:141-8. |  |
| Pinquart M. Self-esteem of children with and without chronic physical illness: a meta-analysis. Child Care Health Dev 2013;39:153-61 |  |
| **reviews not meeting the selection criteria for a systematic review** | **reason(s) for exclusion** |
| Sponseller PD, Yazici M, Demetracopoulos C, Emans JB. Evidence basis for management of spine and chest wall deformities in children. Spine 2007;32:19S:S81-S90 | search details, inclusion and exclusion criteria, quality appraisal of included studies not reported; only types of included studies with levels of evidence stated |
| Tones M, Moss N, Polly DW. A review of quality of life and psychosocial issues in scoliosis. Spine 2006;31:3027–38. | general information on search strategy (databases searched, but no data on keywords or limits) and selection criteria provided; included studies individually summarized, without a quality assessment |
| Sponseller PD. Bracing for adolescent idiopathic scoliosis in practice today. J Pediatr Orthop 2011;31(1 Suppl):S53-60. | critical review – does not meet the criteria for an SR |

Table S2. Cont.

| **reviews not meeting the selection criteria for a systematic review** | **reason(s) for exclusion** |
| --- | --- |
| Weinstein SL, Dolan LA, Cheng JCY, Danielson A, Morcuende JA. Adolescent idiopathic scoliosis. Lancet 2008;371:1527-37. | critical review: general information on search strategy and selection criteria provided; quality assessment of included papers not reported, only sample size of included brace studies addressed |
| Maruyama T. Bracing adolescent idiopathic scoliosis: A systematic review of the literature of effective conservative treatment looking for end results after weaning. Disabil Rehabil 2008;30:786-91. | PubMed/ Medline search performed, but included studies only summarised narratively, without a quality assessment or data synthesis |
| Wong MS, Liu WC. Critical review on non-operative management of adolescent idiopathic scoliosis. Prosthet Orthot Int 2003;27:242-53.** | searching strategy and selection criteria not stated; included studies discussed narratively |
| Hawes MC. The use of exercises in the treatment of scoliosis: an evidence-based critical review of the literature. Pediatr Rehabil 2003;6:171-82.** | searching strategy reported, but included studies only summarised narratively |
| Weiss HR, Werkmann M. Soft braces for the treatment of adolescent idiopathic scoliosis (AIS) – review of the literature and description of a new approach. Scoliosis 2012, 7:11 | search strategy reported, but only keywords provided; quality assessment of included papers – not reported |
| Negrini S. Bracing adolescent idiopathic scoliosis today. Disabil Rehabil Assist Technol 2008;3:107-11 | narrative review and editorial |
| Weiss HR, Goodall D. Is in-patient scoliosis rehabilitation clinically effective: a systematic PubMed review. Scoliosis 2009;4(Suppl 2):O32 | abstract / conference proceedings |
| Le V. Adolescent Idiopathic Scoliosis: Bracing. Joanna Bricks Institute database 2011, ID JBI1592. | evidence summary (publication type) |

Table S2. Cont.

| **reviews not meeting the selection criteria for a systematic review** | **reason(s) for exclusion** |
| --- | --- |
| Schiller JR, Thakur NA, Eberson CP. Brace management in adolescent idiopathic scoliosis. Clin Orthop Relat Res 2010;468:670-78 | the authors report that they included 75 papers for full text analysis, but do not state what studies were finally included; then 11 studies are listed in tables and discussed narratively; no critical appraisal reported; not clear whether the search was comprehensive: “multiple search engines, including Ovid®, MedLine®, and PubMed®” are reported as sources of data, with no time limits stated |
| Weiss H-R. Intervention studies on scoliosis – Review of the reviews. Pol Ann Med 2012;19:72-83 | searching PubMed only; original articles also eligible; no quality appraisal |
| Weiss H-R. Physical therapy intervention studies on idiopathic scoliosis-review with the focus on inclusion criteria. Scoliosis 2012;7:4 | does not meet inclusion criteria for this overview –concentrates on discussing inclusion criteria for primary papers included in the review, not on outcomes measured |
| Stokes OM, Luk KD. The current status of bracing for patients with adolescent idiopathic scoliosis. Bone Joint J 2013;95-B:1308-16 | an “instructional review”; included studies ordered by design and level of evidence, but search and selection strategy, and quality assessment not reported |
| Negrini S, De Mauroy JC, Grivas TB, Knott P, Kotwicki T, Maruyama T, O'Brien JP, Rigo M, Zaina F. Actual evidence in the medical approach to adolescents with idiopathic scoliosis  Eur J Phys Rehabil Med 2014;50:87-92 | critical review |
| Zaina F., De Mauroy J. C., Grivas T., Hresko M. T., Kotwicki T., Maruyama T., Price N., Rigo M., Stikeleather L., Wynne J., Negrini S. [Bracing for scoliosis in 2014: state of the art](http://www.minervamedica.it/en/journals/europa-medicophysica/article.php?cod=R33Y2014N01A0093). Eur J Phys Rehabil Med 2014;50:93-110 | critical review |
| Bettany-Saltikov J, Parent E, Romano M, Villagrasa M, Negrini S. Physiotherapeutic scoliosis-specific exercises for adolescents with idiopathic scoliosis. Eur J Phys Rehabil Med 2014;50:111-21 | critical review |

Table S2. Cont.

| **evidence reviews supporting recommendations/ guidelines** | **reason(s) for exclusion** |
| --- | --- |
| Philadelphia Panel Members. Philadelphia Panel evidence-based clinical practice guidelines on selected rehabilitation interventions: overview and methodology. Phys Ther 2001; 81:1629-40 | scoliosis was an exclusion criterion; basing on this analysis, no further Philadelphia Panel guidelines were analysed |
| [No authors listed]. The management of spinal deformity in the United Kingdom. A guide to good practice. British Orthopaedic Association, 2003. [www.boa.ac.uk](http://www.boa.ac.uk) | not a systematically developed guideline, does not consider nonoperative treatment |
| Britnell SJ, Cole JV, Isherwood L, et al. Postural health in women: the role of physiotherapy. J Obstet Gynaecol Can 2005;27:493-500 | scoliosis not specifically addressed |
| Weiss HR, Negrini S, Hawes MC, et al. Physical exercises in the treatment of idiopathic scoliosis at risk of brace treatment – SOSORT consensus paper 2005. Scoliosis 2006, 1:6 | expert opinion / survey |
| SOSORT guideline committee, Weiss H-R, Negrini S, Rigo M, Kotwicki T, Hawes MC, et al. Indications for conservative management of scoliosis (guidelines). Scoliosis 2006, 1:5. http://www.scoliosisjournal.com/content/1/1/5. | not systematically developed guideline; also: update published |
| Kotwicki T, Durmała J, Czaprowski D, Głowacki M, Kołban M, et al.; SOSORT. Conservative management of idiopathic scoliosis--guidelines based on SOSORT 2006 Consensus. Ortop Traumatol Rehabil. 2009;11:379-95 | document based on the 2006 SOSORT guideline |
| HAS – French national Authority for Health. [Scoliose structurale évolutive (dont l’angle est égal ou supérieur à 25°) jusqu’à maturation rachidienne. Guide ALD](http://www.g-i-n.net/library/international-guidelines-library/guidelines/has-fr/scoliose-structurale-evolutive-dont-l2019angle-est). February 2008. http://www.has-sante.fr/portail/upload/docs/application/pdf/guidem_scoliose_web.pdf | systematic review not reported |
| American Chiropractic Board of Sports Physicians (ACBSP). Appropriateness of physical and sporting activity for those with scoliosis. ACBSP; Estherville (IA): 2009 Jun 5 | duplicate - a separately published SR supporting this guideline included for analysis [J Chiropr Med 2009;8:25-37] |
| Negrini S, Aulisa AG, Aulisa L, et al. 2011 SOSORT guidelines: Orthopaedic and rehabilitation treatment of idiopathic scoliosis during growth. Scoliosis 2012;7:3. | both consensus-, and evidence-based guideline;  systematic search reported; however, inclusion criteria address availability of publications, not study designs; included studies reported narratively; quality assessment not reported |

Table S2. Cont.

| **narrative reviews** |
| --- |
| Farady JA. Current principles in the nonoperative management of structural adolescent idiopathic scoliosis. Phys Ther 1983;63:512-523. |
| Dutro CL, Keene KJ. Electrical muscle stimulation in the treatment of progressive adolescent idiopathic scoliosis: a literature review. J Manipulative Physiol Ther 1985;8:257-260. |
| Rinsky LA, Gamble JG. Adolescent idiopathic scoliosis. West J Med 1988;148:182-191. |
| Keller RB. Nonoperative treatment of adolescent idiopathic scoliosis. Instr Course Lect 1989;38:129-135. |
| Cassella MC, Hall JE. Current treatment approaches in the nonoperative and operative management of adolescent idiopathic scoliosis. Phys Ther 1991;71:897-909. |
| Bergoin M. Treatment of idiopathic scoliosis in children. Ann Pediatr (Paris) 1993;40:259-269. |
| Ebenbichler G, Liederer A, Lack W. Scoliosis and its conservative treatment possibilities. Wien Med Wochenschr 1994;144:593-604. |
| Lonstein JE. Adolescent idiopathic scoliosis. Lancet 1994;344:1407-12. |
| Skaggs DL, Bassett GS. Adolescent idiopathic scoliosis: an update. Am Fam Physician 1996;53:2327-35. |
| Roach JW. Adolescent idiopathic scoliosis. Orthop Clin North Am 1999;30:353-65. |
| Dickson RA. Spinal deformity – adolescent idiopathic scoliosis. Nonoperative treatment. Spine 1999;24:2601-6. |
| Dickson RA, Weinstein SL. Bracing (and screening) – yes or no? J Bone Joint Surg [Br] 1999, 81-B:193-198. |
| Reamy BV, Slakey JB. Adolescent idiopathic scoliosis: review and current concepts. Am Fam Physician 2001;64:111-116. |
| Burgoyne W, Fairbank J. The management of scoliosis. Curr Pediatr 2001;11:323-331. |
| Weiss HR. Rehabilitation of adolescent patients with scoliosis – what do we know? A review of the literature. Pediatr Rehabil 2003:6:183-94 |
| Koumbourlis AC. Scoliosis and the respiratory system. Paed Resp Rev 2006;7:152-60. |
| Asher MA, Burton DC. Adolescent idiopathic scoliosis: natural history and long term treatment effects. Scoliosis 2006;1:2 |
| Lonstein JE. [Scoliosis: surgical versus nonsurgical treatment.](http://www.ncbi.nlm.nih.gov/pubmed/16462448) Clin Orthop Relat Res. 2006;443:248-59 |
| Shelton YA. Scoliosis and kyphosis in adolescents: diagnosis and management. Adolesc Med State Art Rev 2007;18:121-39 |
| Shaughnessy WJ. Advances in scoliosis brace treatment for adolescent idiopathic scoliosis. Orthop Clin North Am 2007;38:469-75 |
| Schiller JR, Eberson CP. Spinal deformity and athletics. Sports Med Arthrosc 2008;16:26-31 |
| Zarzycka M, Rozek K, Zarzycki M. Alternative methods of conservative treatment of idiopathic scoliosis. Ortop Traumatol Rehabil 2009;11:396 |
| Kim HJ, Blanco JS, Widmann RF. Update on the management of idiopathic scoliosis. Curr Opin Pediatr 2009;21:55-64. |
| Fayssoux RS, Cho RH, Herman MJ. A history of bracing for idiopathic scoliosis in North America. Clin Orthop Relat Res 2010;468:654-64 |
| Rigo MD, Grivas TB. "Rehabilitation schools for scoliosis" thematic series: describing the methods and results. Scoliosis 2010;5:27. |
| Trobisch P, Suess O, Schwab F. Idiopathic scoliosis. Dtsch Arztebl Int 2010;107:875-84. |
| Weiss HR. Spinal deformities rehabilitation - state of the art review. Scoliosis 2010;5:28 |

Table S2. Cont.

| **narrative reviews** | |
| --- | --- |
| Diokno E, Rowe D. Medical and orthopedic conditions and sports participation. Pediatr Clin North Am 2010;57:839-47 | |
| Canavese F, Kaelin A. Adolescent idiopathic scoliosis: Indications and efficacy of nonoperative treatment. Indian J Orthop 2011;45:7-14. | |
| Sud A, Tsirikos AI. Current concepts and controversies on adolescent idiopathic scoliosis: Part I. Indian J Orthop 2013;47:117-128 | |
| Hoashi JS, Cahill PJ, Bennett JT, Samdani AF. [Adolescent scoliosis classification and treatment.](http://www.ncbi.nlm.nih.gov/pubmed/23561556) Neurosurg Clin N Am 2013;24:173-83 | |
| Schlenzka D, Yrjönen T. Bracing in adolescent idiopathic scoliosis. J Child Orthop 2013;7:51-5 | |
| Hresco MT. Idiopathic scoliosis in adolescents. N Engl J Med 2013;368:834-41 | |
| Altaf F, Gibson A, Dannawi Z, Noordeen H. Adolescent idiopathic scoliosis. BMJ 2013;346:f2508 | |
| Falk B, Rigby A, Akseer N. Adolescent idiopathic scoliosis: the possible harm of bracing and the likely benefit of exercise. Spine J 2014; DOI 10.1016/j.spinee.2014.05.006 | |
| **other papers** | **reason(s) for exclusion** |
| Maruyama T, Kitagawa T, Takeshita K, Mochizuki K, Nakamura K. Conservative treatment of adolescent idiopathic scoliosis: can it reduce the incidence of surgical treatment? Pediatric Rehabil 2003;3-4:215-9 | primary study |
| Farley R, Clark J, Davidson C. What is the evidence for the effectiveness of postural management? Int J Ther Rehabil 2003;10:449-55 | idiopathic scoliosis not an inclusion criterion |
| Weiss HR. Conservative treatment of scoliosis. Pediatric Rehabil 2003;3-4:131-2 | editorial |
| Weiss HR. Is there a body of evidence for the treatment of patients with adolescent idiopathic scoliosis (AIS)? Scoliosis 2007;2:19 | editorial |
| Negrini S, Atanasio S, Zaina F, Romano M. Rehabilitation of adolescent idiopathic scoliosis: results of exercises and bracing from a series of clinical studies. Eur J Phys Rehabil Med 2008;2:169-76 | duplicate: the paper summarises three SRs [51-53], analysed individually in the paper |
| Negrini S. Approach to scoliosis changed due to causes other than evidence: patients call for conservative (rehabilitation) experts to join in team orthopedic surgeons. Disabil Rehabil 2008;30:731-41 | bibliometric study with a narrative review, and case descriptions |
| Kotwicki T. Evaluation of scoliosis today: examination, X-rays and beyond. Disabil Rehabil 2008;30:742-51 | narrative review, not on treatment effectiveness |
| Smania N, Picelli A, Romano M, Negrini S. Neurophysiological basis of rehabilitation of adolescent idiopathic scoliosis. Disabil Rehabil 2008;30:763-71 | not an intervention study |

Table S2. Cont.

| **other papers** | **reason(s) for exclusion** |
| --- | --- |
| Bagnall KM. Using a synthesis of the research literature related to the aetiology of adolescent idiopathic scoliosis to provide ideas on future directions for success. Scoliosis 2008, 3:5 | narrative review / expert opinion |
| Goldberg CJ, Moore DP, Fogarty EE, Dowling FE. Scoliosis: a review. Pediatr Surg Int 2008;24:129-44 | original study with an extended narrative discussion of the literature |
| Holzapfel BM, Prodinger PM, Burklein D, Rudert M, Mrosek EH, Mayer-Wagner S. [Conservative treatment for adolescent idiopathic scoliosis]. MMW Fortschr Med 2010; 15:34-6 | full text not retrieved |
| Weiss HR. Scoliosis and Evidence-based practice. Physiother Theor Pract 2011;27:2-6. | editorial |
| Weiss HR. Inclusion criteria for physical therapy intervention studies on scoliosis – a review of the literature. Stud Health Technol Inform 2012;176:350-3 | duplicate: second report from the same study [Scoliosis 2012;7:4] |
| Weiss HR, Moramarco M. Indication for surgical treatment in patients with adolescent Idiopathic Scoliosis - a critical appraisal. Patient Saf Surg. 2013;7:17 | letter to the editor |

*Vitale MA, Vitale MG, Zivin JG, et al. Rotator cuff repair: an analysis of utility scores and cost-effectiveness. J Shoulder Elbow Surg 2007;16:181–7: “Preoperative rHuEPO transfusion versus PAD in 15-year-old female undergoing surgery for adolescent scoliosis”; ** due to variations in terminology, papers entitled “critical review” were not automatically excluded
