# Supplementary material for: Non-Surgical Interventions for Adolescents with Idiopathic Scoliosis: An Overview of Systematic Reviews
Source: PLoS One. 2014 Oct 29;9(10):e110254. doi: 10.1371/journal.pone.0110254 (PMC4213139; doi:10.1371/journal.pone.0110254)
Supplement: Table S3 — Details of the electronic search and selection process. (DOCX) [file pone.0110254.s003.docx]

Table S3. Details of the electronic search and selection process.

| database/ source | date of search | query time span | search terms/ limits | records found/  selected for full text analysis/  included |
| --- | --- | --- | --- | --- |
| databases of systematic reviews, databases with separate indexing of systematic reviews, guideline registries | | | | |
| Cochrane CDSR database | 15 03 2014 | unlimited | scoliosis | 6/2/2 |
| Centre for Reviews and Dissemination: |  |  |  |  |
| DARE | 15 03 2014 | unlimited | scoliosis | 47/ 5/5 |
| HTA | 15 03 2014 | unlimited | scoliosis | 13/2/0 |
| NHSEED | 15 03 2014 | unlimited | scoliosis | 24/0/0 |
| Joanna Briggs Institute: |  |  |  |  |
| Database of Systematic Reviews and Implementation Reports | 16 03 2014 | unlimited | scoliosis | 5/2/0 |
| JBI COnNECT+ database |  |  | scoliosis;  limits: systematic reviews | 2/1/0 |
| PEDro | 16 03 2014 | unlimited | scoliosis;  limits: systematic reviews,  limits: guidelines | 19/10/7  1/1/0 |
| National Guideline Clearinghouse |  | unlimited | scoliosis | 25/0/0 |
| TRIP | 16 03 2014 | unlimited | idiopathic scoliosis  refinement: systematic reviews  refinement: guidelines | 11/0/0  28/1/0 |
| Campbell Library | 16 03 2014 | unlimited | scoliosis | 0/0/0 |
| websites of institutions | | | | |
| Scoliosis Research Society (SRS) | 16 03 2014 | n/a | website searched for relevant publications | 1/ 0/0 |
| Society for Spinal Orthopaedic and Rehabilitation Treatment (SOSORT) | 16 03 2014 | unlimited | “Scoliosis” journal browsed for SOSORT guidelines | 3/3/0 |
| International Research Society for Spinal Deformities (IRSSD) | 16 03 2014 | n/a | website searched for relevant publications | 0/0/0 |
| Guidelines International Network (G-I-N) | 16 03 2014 | unlimited | scoliosis | 4/1/0 |

Table S3. Cont.

| database/ source | date of search | query time span | search terms/ limits | records found/  selected for full text analysis/  included |
| --- | --- | --- | --- | --- |
| Scottish Intercollegiate Guideline Network (SIGN) | 16 03 2014 | unlimited | whole guideline list browsed | 0/0/0 |
| National Institute for Clinical Excellence (NICE), UK | 16 03 2014 | unlimited | scoliosis | 15/ 0/0 |
| Agency for Healthcare Research and Quality (AHRQ)/ Evidence-based Practice Centres: Evidence-based Reports | 16 03 2014 | current reports | A-Z topic index | 0/0/0 |
| National Health and Medical Research Council, Australia (NHMRC) | 16 03 2014 | unlimited | scoliosis | 0/0/0 |
| general bibliographic databases | | | | |
| MEDLINE/ PubMed | 19 03 2014 | publication dates: 1980-latest available | (idiopathic[All Fields] AND ("scoliosis"[MeSH Terms] OR "scoliosis"[All Fields])) AND (Meta-Analysis[ptyp] OR systematic[sb] OR Guideline[ptyp]) | 108/18/4 |
| Web of Science: Science Citation Index – EXPANDED (SCI – EXPANDED) | 19 03 2014 | timespan=1980-latest available | #1 TS=(idiopathic) AND TS=(scoliosis) AND TS=(systematic review)  #2 TS=(idiopathic) AND TS=(scoliosis) AND TS=(meta-analysis)  #3 TS=(idiopathic) AND TS=(scoliosis) AND TS=(guideline)  #4 #3 OR #2 OR #1 | -  -  -  138/ 2/ 0 |
| SportsDiscus | 31 03 2014 | 2001 – 31 12 2013 | scoliosis AND idiopathic AND systematic reviews | 50/ 3/ 1 |
