# Supplementary material for: Non-Surgical Interventions for Adolescents with Idiopathic Scoliosis: An Overview of Systematic Reviews
Source: PLoS One. 2014 Oct 29;9(10):e110254. doi: 10.1371/journal.pone.0110254 (PMC4213139; doi:10.1371/journal.pone.0110254)
Supplement: Protocol S1 — PROSPERO protocol. (PDF) [file pone.0110254.s004.pdf]

Effects of nonsurgical management for patients with idiopathic scoliosis: an overview of systematic reviews

Maciej Plaszewski, Josette Bettany-Saltikov

Citation

Maciej Plaszewski, Josette Bettany-Saltikov. Effects of nonsurgical management for patients with idiopathic scoliosis: an overview of systematic reviews. PROSPERO 2013:CRD42013003538 Available from
http://www.crd.york.ac.uk/PROSPERO_REBRANDING/display_record.asp?ID=CRD42013003538

Review question(s)

To summarise evidence for the effectiveness of conservative therapies and screening for idiopathic scoliosis.

To assess the quality of published systematic reviews of these interventions.

To find which systematic reviews of the same interventions formulate more reliable conclusions.

Searches

Databases searched:

MEDLINE usingPubMed, SportsDiscus using EBSCOhost Web, and Web of Science, Cochrane Database of Systematic Reviews (CDSR), Database of Abstracts of Reviews of Effectiveness (DARE), NHS Economic Evaluation Database (NHSEED), HTA Database, Physiotherapy Evidence Database (PEDro), the Campbell Collaboration Library of Systematic Reviews (the Campbell Library), the Joanna Briggs Institute and the TRIP database, directly at their sites.

Searching for protocols and registered titles:

PROSPERO, Cochrane Library, Campbell Library, Joanna Briggs Institute and the Systematic Reviews journal are searched for relevant registered protocols or titles.

Handsearching, reference checking, and other:

We handsearched reference lists of relevant papers. The National Guideline Clearinghouse database is searched and, if relevant clinical practice guidelines are found, their reference lists are handsearched.

Restrictions:

Only systematic reviews published in full texts are included for analysis. We do not contact potential authors of ongoing unpublished and not registered systematic reviews. Titles and abstracts published in English language are considered. If found, full texts of articles in a different language will be translated. Databases will be searched from inception up to December 1, 2012, with subsequent updating searches. Two searchers independently conduct the database search, and the selection of included titles, abstracts, and, if necessary, full text articles. If needed, decisions upon inclusion or exclusion of selected papers will be reached by consensus.

Link to search strategy

http://www.crd.york.ac.uk/PROSPEROFILES/3538_STRATEGY_20140605.pdf

Types of study to be included

Inclusion criteria:

Included are papers meeting main criteria for a systematic review: clear inclusion/exclusion criteria, an explicit search

strategy, and systematic analysis of included studies. Both quantitative and qualitative (without a meta-analysis) systematic reviews of any types of primary experimental or observational studies are included.

Exclusion criteria:

Narrative and other types of non-systematic reviews (e.g. critical reviews, overviews, state-of-the-art reviews), clinical practice guidelines, evidence summaries, critically appraised topics, clinical paths, consumer information sheets, best practice information sheets, technical reports, and other evidence-based pieces, are excluded.

Condition or domain being studied

Idiopathic scoliosis.

Scoliosis is the most prevalent orthopaedic condition affecting children. In its most frequent type, the adolescent idiopathic scoliosis, is diagnosed in about 80% of cases of this condition in patients of pubertal age, mostly in girls. The risk of progression depends on skeletal maturity, curve size, and gender. Idiopathic scoliosis may have lasting consequences and can be accompanied with pulmonary complications, pain symptoms, difficulties in participation, and psychological disorders. Expert opinions and study findings regarding therapeutic interventions and screening are inconsistent.

Participants/ population

Reviews regarding adolescents with idiopathic scoliosis are eligible.

Intervention(s), exposure(s)

Inclusion criteria:

Systematic reviews of screening for scoliosis, and of conservative treatments (bracing, exercise therapy, observation, and any other non-operative approaches) are eligible.

Exclusion criteria:

Excluded are systematic reviews of surgical treatments and of diagnostic approaches (e.g. x-ray) other than those used in screening procedures. Systematic reviews regarding mixed populations (e.g. of low back pain in various conditions, including idiopathic scoliosis) are also ineligible.

Comparator(s)/ control

There is no restriction for control interventions. It may be no intervention or other type(s) of intervention(s) (e.g. different types of braces, exercise treatments, or electrical stimulation), depending on what control interventions were considered in included systematic reviews.

Context

We do not restrict our eligibility criteria to any context. We include studies regarding interventions delivered outside the health care setting (e.g. brace treatment), and in public and private practice (e.g. inpatient and outpatient physiotherapy or rehabilitation programmes), and school screening.

Outcome(s)

Primary outcomes

Any outcomes related to objective functioning and subjective wellbeing regarding screening and treatment effectiveness, e.g. curve progression, trunk rotation, physical functioning, body image, self-acceptance, and health-related quality of life, are considered.

Both short-term and long-term outcomes are considered, depending on the characteristics of individual systematic reviews.

Secondary outcomes

There is no restriction for secondary outcomes.

Data extraction, (selection and coding)
We will independently carry out study inclusion and coding decisions. If present, uncertainties are resolved by discussion and consensus. This can be done at the initial step of eligibility screening based on titles and abstracts, but also, if necessary, by full text analyses. Both searchers follow explicitly the study protocol and base the selection process on a search strategy formulated with a collaborating experienced biomedical librarian.

Risk of bias (quality) assessment
We will independently assess the quality of included studies with the AMSTAR tool for quality assessment of systematic reviews. Inter-rater reliability will be assessed with kappa and, if required, a collaborator will be invited to conduct further appraisals.

Strategy for data synthesis
It is not planned to subject the data to meta-analysis. A descriptive synthesis of assessed individual systematic reviews is planned. Excluded papers will be listed, with reasons for exclusion stated.

Analysis of subgroups or subsets
None planned.

Contact details for further information
Maciej Plaszewski

University School of Physical Education,

Faculty of Physical Education and Sport,

Institute of Physiotherapy,

Akademicka 2

21-500 Biala Podlaska,

Poland

plaszewski@wp.pl

Organisational affiliation of the review
Warsaw University School of Physical Education, Faculty of Physical Education and Sport in Biala Podlaska, Institute of Physiotherapy

www.awf-bp.edu.pl

Review team
Dr Maciej Plaszewski, Warsaw University School of Physical Education, Faculty of Physical Education and Sport in Biala Podlaska, Institute of Physiotherapy
Dr Josette Bettany-Saltikov, School of Health and Social Care, University of Teesside, Middlesbrough, UK

Collaborators
Dr Igor Cieslinski, Warsaw University School of Physical Education, Faculty of Physical Education and Sport in Biala Podlaska, Poland

Details of any existing review of the same topic by the same authors
This is a new review.

Anticipated or actual start date
15 October 2012

Anticipated completion date
10 July 2014

Funding sources/sponsors
None.

Conflicts of interest
None known

Language
English

Country
Poland

Subject index terms status
Subject indexing assigned by CRD

Subject index terms
Humans; Orthopedic Procedures; Quality of Life; Scoliosis

Any other information
Part of the review, regarding screening, has been published. The second report, regarding treatment interventions, has been completed, but not published

Stage of review
Completed but not published

Date of registration in PROSPERO
02 January 2013

Date of publication of this revision
07 July 2014

Details of final report/publication(s)
http://link.springer.com/article/10.1007%2Fs00586-014-3307-x

Stage of review at time of this submission	Started	Completed
Preliminary searches	No	Yes
Piloting of the study selection process	No	Yes
Formal screening of search results against eligibility criteria	No	Yes
Data extraction	No	Yes
Risk of bias (quality) assessment	No	Yes
Data analysis	No	Yes

PROSPERO
International prospective register of systematic reviews
The information in this record has been provided by the named contact for this review. CRD has accepted this information in good faith and registered the review in PROSPERO. CRD bears no responsibility or liability for the content of this registration record, any associated files or external websites.
